# Orogeny and High Pollen Flow as Driving Forces for High Genetic Diversity of Endangered *Acer griseum* (Franch.) Pax Endemic to China

**DOI:** 10.3390/ijms26020574

**Published:** 2025-01-11

**Authors:** Xinhe Xia, Xuedan Yu, Yuxia Wu, Jia Liao, Xinyue Pan, Yongqi Zheng, Chuanhong Zhang

**Affiliations:** State Key Laboratory of Tree Genetics and Breeding, Research Institute of Forestry, Chinese Academy of Forestry, Beijing 100091, China; xinhex355@163.com (X.X.); yuxd@caf.ac.cn (X.Y.); wuyux3@163.com (Y.W.); liaojiaa23@163.com (J.L.); 13397144839@163.com (X.P.)

**Keywords:** genetic diversity, phylogeography, *Acer griseum*, orogeny, environmental stress, gene flow

## Abstract

*Acer griseum* (Franch.) Pax is an endangered species endemic to China, mainly scattered in the Qinling–Daba Mountains. The genetic diversity of 17 natural populations were analyzed by nuclear DNA (nDNA) and chloroplast DNA (cpDNA) to explore the driving forces for its microevolution. A high level of genetic diversity (nDNA: *H*e = 0.296, cpDNA: *H*t = 0.806) was found in *A. griseum*. Genetic variation was mainly within populations (92.52%) based on nDNA, while it was mainly among populations (96.26%) based on cpDNA. The seventeen populations were divided into two groups, corresponding to the subtropical zone (Group I) and temperate zone (Group II), with haplotype 4 (Hap4) and Hap5 being the most common haplotypes, respectively. Consequently, genes associated with heat and heavy metal stress were identified in Group I, while genes related to salt and drought stress were identified in Group II. Haplotype differentiation was driven by the heterogeneous microenvironment caused by the uplifting of the Qinling-Daba Mountains, which was a vital source of its high genetic diversity. Furthermore, the uplifted Qinling–Daba mountains may bridge high pollen flow among populations, whereas rivers can result in low seed flow among populations, which has led to the incongruent genetic structure between nDNA and cpDNA. This study represents a new perspective that geological events, especially orogeny, play an important role in plant microevolution through the establishment of maternal genetic structure and provides a meaningful conservation strategy for *A. griseum*. Overall, the Qinling–Daba Mountains not only are cradles for the genetic diversity of *A. griseum* but also provided refugia for it during the Quaternary glacial period.

## 1. Introduction

Mountains play an important role in the conservation and maintenance of plant genetic diversity. Firstly, mountains act as cradles for biodiversity [1,2]. In China, both the Hengduan Mountains and Qinling–Daba Mountains are world-famous for harboring abundant biotas with many ancient and endemic species, such as *Acer* L. species [3,4,5]. Grand mountains can lead to the differentiation of climatic zones, with the consequence of driving plant population divergence, such as how the Qinling Mountains separate the temperate zone from the subtropical zone in Central China. Orogeny also can lead to heterogeneous microenvironments or microterrains, favoring the rapid in situ speciation of indigenous lineages [6,7]. Because of maternally inherited characteristics with independent evolutionary routes for angiosperm, chloroplast DNA (cpDNA) can be used to reveal the spatio-temporal dynamics of plant populations promoted by orogeny [8]. Secondly, mountains have acted as refugia for plants throughout their long evolutionary history, shielding them from climate oscillations and conserving genetic diversity. Although China was less influenced by the Quaternary glaciation than Europe, the drastic climate oscillation still led to the migration or adaptive evolution of many local species [9,10]. Due to the complexity of their climate and topography, the Qinling–Daba Mountains have acted as refugia, providing shelters for many endemic and endangered plants throughout the Quaternary, such as *Dipteronia sinensis* Oliv., *Pinus henryi* Mast., *Quercus baronii* Skan, and *Q. dolicholepis* A. Camus [11,12,13]. Species distribution models (SDMs) are widely used to analyze the impact of historical and future climate-induced fluctuation on the potential distribution of target species and can analyze demographic histories and propose future innovative conservation strategies for genetic resources from a new perspective [14]. Thirdly, mountains and rivers can be bridges or barriers of gene flow among populations of a species [15,16] and drive species evolution [17], population divergence [18], and haplotype differentiation [19] in many plant species. Pollen plays a major role in connecting extant populations with gene flow, but seeds are necessary to the colonization of plants [8]. It becomes possible to identify the role of both pollen-mediated and seed-mediated gene flow in shaping an extant spatial population of plants’ genetic structure by using nuclear DNA (nDNA) and cpDNA [20,21,22], such as *Quercus aliena* Blume [23] and *Parrotia subaequalis* (H. T. Chang) R. M. Hao and H. T. Wei [24]. Genetic diversity is crucial for the conservation of endangered plants [25,26,27] and is jointly influenced by external factors (orogeny, climatic oscillation, etc.) and internal factors (gene flow, mating system, etc.) [28,29]. Thus, it is of great significance for understanding how orogeny, climate oscillation, and gene flow are separately or jointly involved in the evolutionary process of genetic diversity and spatial genetic structure of plants, especially for endangered plants.

**Figure 1:**
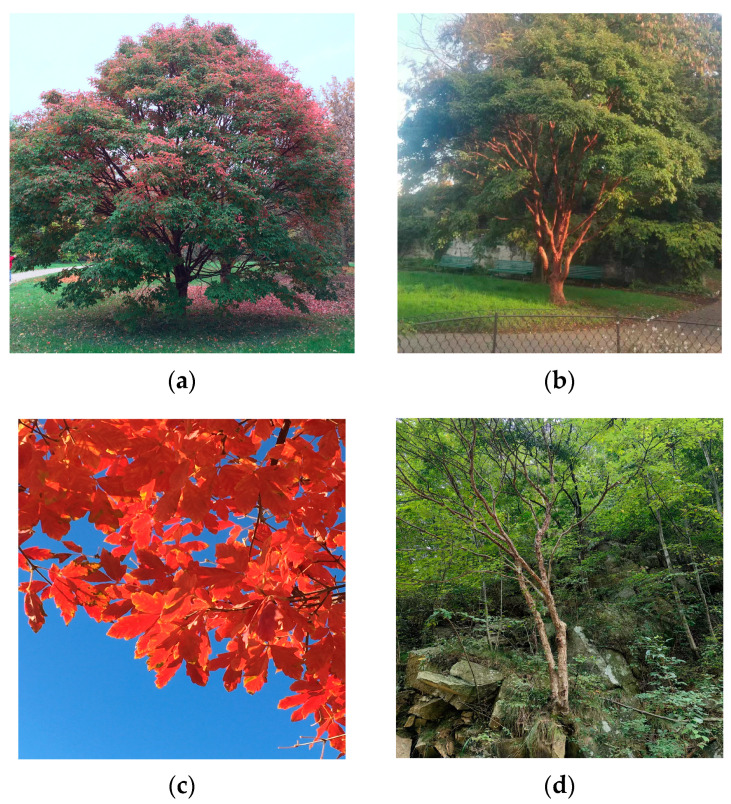
Panorama of *A. griseum*. (**a**) The individual of *A. griseum* introduced to the Botanical Garden in Geneva, Switzerland, showed good growth and striking colorful leaves in autumn. (**b**) *A. griseum* planted in garden near Geneva Lake in Switzerland with conspicuous reddish-brown trunk in contrast with green leaves. (**c**) *A. griseum* planted in Chinese Academy of Forestry in Beijing with scarlet trifoliate foliage. (**d**) *A. griseum* grows on bare rock in one of its natural habitats, indicating its strong resistance to barren terrain.

*Acer griseum* (Franch.) Pax (Sapindaceae Juss.) is an endangered tree species endemic to China, mainly distributed at an altitude of 800–1800 m as a subdominant tree species on steep slopes and cliffs in four mountains (Mt. Taihang, Mt. Qinling, Mt. Daba, and Mt. Wuling) spanning subtropical and temperate zones. It is of high ornamental value, not only because of its magnificent papery peeling, cinnamon to reddish-brown bark, but also because of its striking scarlet trifoliate foliage in autumn (Figure 1a–c). It can also serve as a prime candidate for specimen planting in gardens and residential landscape. Moreover, it is also a precious timber tree due to its hard wood. It was introduced to Europe by Ernest Henry Wilson from China in 1901 and then introduced to America in 1907. Until now, it has been planted in famous arboretums such as Arnold Arboretum and Kew Gardens [30]. At the end of the last century, it began to be cultivated in Beijing, Nanjing, and other cities in China. The Chinese government nominated *A. griseum* as a precious timber tree in 2017. As one of nine compound-leaf maples, *A. griseum* is of high value for scientific studies [31]. Investigations of natural populations found that strong inter-specific competition between *A. griseum* with local dominant species in natural habitat, less fruit bearing of mature trees, and natural regeneration difficulty combined with human logging has led to a gradual reduction of its natural populations [32,33]. Therefore, *A. griseum* has been categorized as an endangered species [34,35] and nowadays understanding the driving forces for its genetic diversity is of top priority for developing a genetic resources conservation strategy for the endangered *A. griseum*.

In general, endangered plants present with low genetic diversity due to inbreeding depression and genetic drift within populations resulted from a small effective population size and limited gene flow among populations [36]. However, there are many endangered plants with high genetic diversity, such as *Trailliaedoxa gracilis* W. W. Sm. and Forrest, *Saussurea involucrata* (Kar. and Kir.) Sch. Bip., and *Camellia tunghinensis* Hung T. Chang [37,38,39,40]. Understanding the driving forces for genetic diversity is essential for the conservation of endangered species. The genetic origin of *A. griseum* from 12 public gardens in the USA and U.K. was analyzed and indicated that they originated from the old big tree planted in Dyffryn Gardens by Ernest Henry Wilson in 1901 [41]. A high level of genetic diversity (*H*e = 0.727) was detected in 11 populations of *A. griseum* based on SSR markers, indicating it is higher than the mean value (*H*e = 0.650) of other perennial plants based on SSR markers [42,43]. However, as an endangered species, how it gained and maintained high genetic diversity and how it shaped its extent spatial genetic structure with the uplift of mountains and climatic oscillation of glacial period are unknown. Given that the genome of *A. griseum* remains unsequenced, its genetic diversity and genetic structure were analyzed by specific length amplified fragment (SLAF) sequencing [22]. Due to its conserved chloroplast genome, polymorphism of universal primers was not detected in *A. griseum* [33]. Complete chloroplast genome sequencing help to develop specific primers to overcome this obstacle [24]. Therefore, the genetic diversity and spatial genetic structure of *A. griseum* were analyzed through nDNA and cpDNA, with the aims of (i) analyzing the driving forces for its genetic diversity, (ii) disclosing what shaped its extant genetic structure, and (iii) exploring the role of orogeny, climate oscillation, and gene flow in shaping its genetic diversity and genetic structure.

## 2. Results

### 2.1. Genetic Diversity and Genetic Differentiation Based on nDNA

#### 2.1.1. Specific-Locus Amplified Fragment Sequencing (SLAF-Seq) Data Quality

A total of 1123.45 Mb reads were obtained from 170 individuals of *A. griseum*, with an average Q30 of 92.90% and an average GC content of 39.13%. The average alignment rate of 170 individuals to the reference genome was 87.28%, with an average sequencing depth of 17.82×. A total of 4,183,022 SNPs (Dataset 1) were identified. After filtering, 271,301 SNPs were retained as Dataset 2, while 92,820 SNPs were retained as Dataset 3.

#### 2.1.2. Genetic Diversity Based on nDNA

The values of proportion of the expected heterozygosity (*H*e), observed heterozygosity (*H*o), Nei’s gene diversity (*H*), Shannon’s information index (*I*), Polymorphism information content (*PIC*), and nucleotide diversity (*π*) in 17 populations ranged from 0.286~0.306, 0.304~0.365, 0.305~0.325, 0.448~0.471, 0.236~0.249, and 0.217~0.252, respectively. There was no great difference in the genetic diversity among the 17 populations. The value of *H*o was greater than *H*e in all populations, indicating that there was no heterozygote loss in *A. griseum*. In addition, inbreeding coefficient (*F*is) in all populations were negative, indicating that there was no inbreeding within the populations (Table S1).

#### 2.1.3. Extant Spatial Genetic Structure

**Figure 2:**
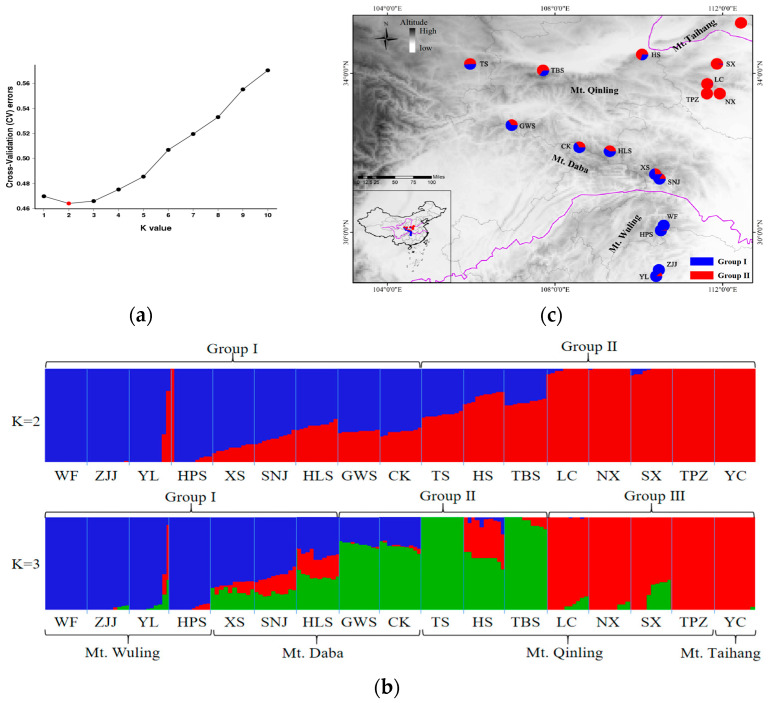
Genetic structure analysis of 17 populations in *A. griseum*. (**a**) Cross-validation (CV) errors for different K values in admixture. (**b**) Clustering analysis results of 17 populations in *A. griseum* by admixture (K = 2 and K = 3). (**c**) Clustering analysis results of 17 populations in *A. griseum* based on their geographic distribution (K = 2).

An admixture analysis of *A. griseum* showed that the CV error value was the lowest when K = 2 (Figure 2a) and the 17 populations were divided into two groups (Figure 2b): the southern group (Group I) and northern group (Group II). Group I consisted of nine populations distributed in Mt. Daba and Mt. Wuling in a subtropical zone, while Group II consisted of eight populations distributed in Mt. Qinling and Mt. Taihang in a temperate zone (Figure 2b). It is worth noting that the genetic components of the populations located in Mt. Taihang, eastern Mt. Qinling, and Mt. Wuling (south and north of its distribution) were relatively homologous, while those in western Mt. Qinling and Mt. Daba (the center of its distribution) were more heterogeneous (Figure 2c). When K = 3, the 17 populations were divided into northern (LC, NX, SX, TPZ, and YC), western (CK, GWS, TS, HS, and TBS), and southern (HLS, WF, ZJJ, YL, HPS, XS, and SNJ) groups. Principal component analysis (PCA) analysis was in agreement with the admixture analysis and revealed that the 17 populations of *A. griseum* were mainly clustered into two genetic groups. Group I consisted of nine populations in the subtropical zone and Group II consisted of eight populations in the temperate zone (Figure S1).

#### 2.1.4. Genetic Differentiation and Gene Flow Among Populations

The Mantel correlation test showed that there was no significant correlation between genetic distance and geographical distance (r = 0.129, *p* > 0.05) (Figure S2). Analysis of molecular variance (AMOVA) using nDNA indicated that its genetic variation was mainly within populations (92.52%), with moderate genetic differentiation (*F*st = 0.075) and high inter-population gene flow (*N*m = 3.083) (Table 1). In addition, the *F*st value among the four mountains varied from 0.032 to 0.094. The gene flow between Mt. Wuling and Mt. Daba was the highest (*N*m = 7.488), followed by the gene flow between Mt. Daba and Mt. Qinling (*N*m = 7.358), and the gene flow between Mt. Wuling and Mt. Taihang was the lowest (2.398) (Table S2). According to TreeMix analysis, gene flow had occurred between populations from Mt. Taihang and Mt. Daba in history (Figure S3).

#### 2.1.5. Phylogenetic Relationship and Divergence Time

**Figure 3:**
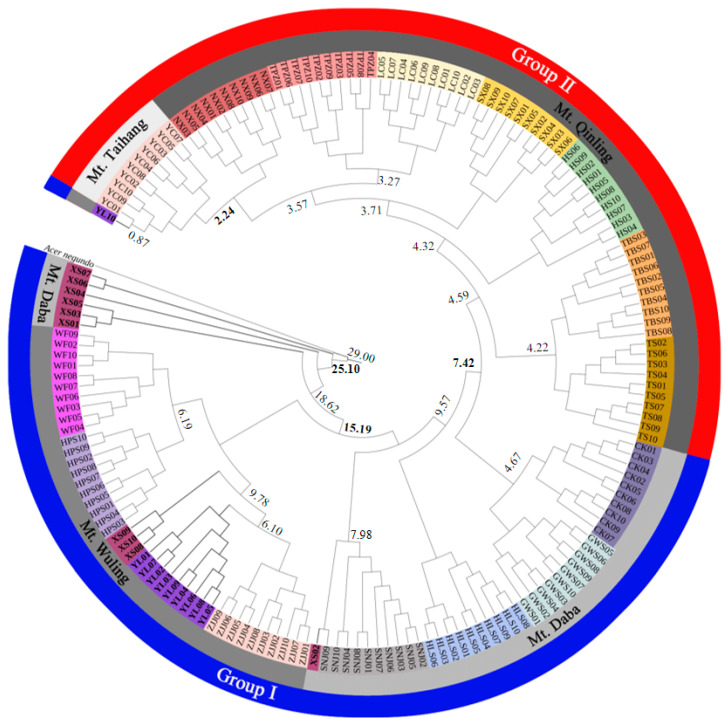
Phylogenetic tree and divergence time of 17 populations in *A. griseum* using the maximum-likelihood (ML) method, with *A. negundo* as an outgroup.

Phylogenetic analysis showed that 15 populations were clustered into monophyly except for the XS and YL populations. In addition, the populations located in the same mountain were primarily clustered together. The populations in Mt. Taihang were found to be closely genetic related with those in Mt. Qinling, while populations in Mt. Wuling were closely genetic related with those in Mt. Daba (Figure 3). The divergence time indicated that the XS population located in Mt. Daba diverged 25.10 million years ago (Mya). At 15.19 Mya, the populations located in Mt. Wuling diverged from the rest of the populations in Mt. Daba. Then, the populations located in Mt. Qinling diverged at 7.42 Mya, while the populations located in Mt. Taihang diverged at 2.24 Mya (Figure 3).

#### 2.1.6. Selective Sweep Analysis and Putative Selected Gene Function Enrichment Analysis

A total of 95 putative selective regions, including 510 genes, were screened and identified in Group I, located in the subtropical zone (Figure S4). Gene Ontology (GO) enrichment analysis showed that 24 GO functional items (*p* < 0.01) were significantly enriched, of which 20 belonged to biological process and four belonged to molecular function. The most significant term was transferase activity, transferring acyl groups other than amino-acyl groups (GO:0016747), followed by male gamete generation (GO:0048232) (Table S3). Based on the Kyoto Encyclopedia of Genes and Genomes (KEGG) database, 28 genes were significantly enriched to five functional items (*p* < 0.01): flavone and flavonol biosynthesis (7), isoflavonoid biosynthesis (7), fatty acid elongation (5), mismatch repair (5), and autophagy—other (4) (Table S4).

A total of 106 putative selective regions, including 451 genes, were screened and identified in Group II, located in the temperate zone (Figure S4). GO enrichment analysis showed that 43 GO functional items (*p* < 0.01) were significantly enriched, of which 39 belonged to biological process, one belonged to cellular components, and three belonged to molecular function. The most significant term was the response to hydrogen peroxide (GO:0042542), followed by UDP-glycosyltransferase activity (GO:0008194) (Table S3). Based on the KEGG database, 46 genes were significantly enriched to five functional items (*p* < 0.01): zeatin biosynthesis (12), phosphatidylinositol signaling system (7), endocytosis (13), inositol phosphate metabolism (7), and pyruvate metabolism (7) (Table S4).

### 2.2. Genetic Diversity and Genetic Differentiation Based on cpDNA

#### 2.2.1. Polymorphic Loci and cpDNA Primers

A total of 121 polymorphic loci were identified by 26 chloroplast genome sequences alignment, of which 52 were SNPs and 69 were indels. Due to the extremely sparse distribution of SNPs, three pairs of primers were finally designed, including a total of 11 polymorphic SNPs (Table S6).

#### 2.2.2. Genetic Diversity Based on cpDNA

The genetic diversity at species level (*H*t) was 0.806, but the genetic diversity at population level (*H*s) was only 0.077. At species level, the haplotype diversity (*H*d) was 0.761 and total nucleotide diversity (*π*) was 0.00118. The highest value of 0.476 in Hd was found in HS population, while the highest value of 0.00024 in *π* was in YL population. Among the four mountains, the highest genetic diversity (*H*d: 0.766, *π*: 0.00144) was found in Mt. Daba, followed by Mt. Qinling, Mt. Wuling, and Mt. Taihang (Table S7).

#### 2.2.3. Genetic Differentiated and Gene Flow Mediated by Seeds

AMOVA analysis using cpDNA showed that the genetic variation was mainly among populations (96.26%), with high genetic differentiation (*F*st = 0.963) and low inter-population seed-mediated gene flow (*N*m = 0.019) (Table 1). In addition, it was found that the *F*st value among the four mountains ranged from 0.313 to 0.980. The seed-mediated gene flow between Mt. Taihang and Mt. Qinling was the highest (*N*m = 1.097), followed by that between Mt. Daba and Mt. Qinling (*N*m = 0.925), and that between Mt. Wuling and Mt. Taihang was the lowest (*N*m = 0.010) (Table S8).

#### 2.2.4. Haplotype Network and Phylogenetic Relationship

**Figure 4:**
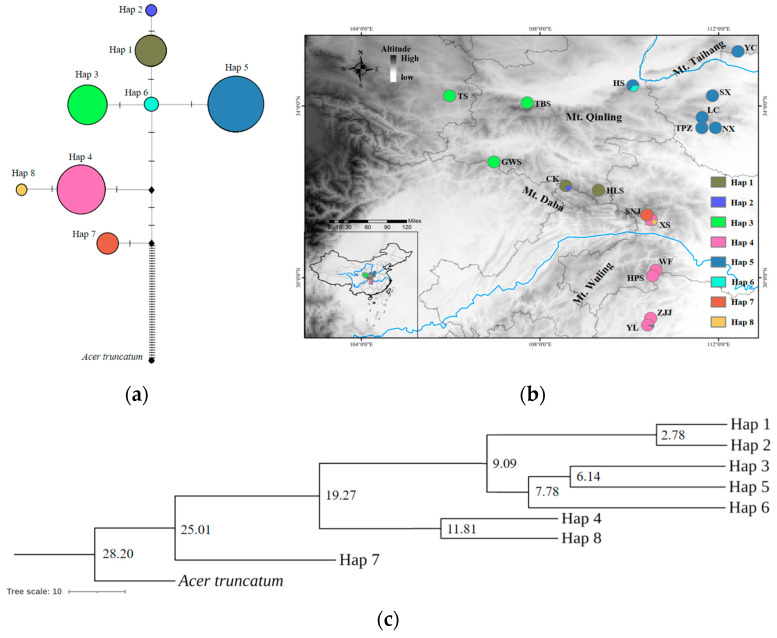
Haplotype network and divergence time of *A. griseum*. (**a**) Haplotype network map of *A. griseum*. (**b**) Geographical distribution of the eight haplotypes identified here, with one solid line between two haplotypes representing one mutational step. (**c**) Divergence time of eight haplotypes of *A. griseum*.

A total of eight haplotypes were identified, of which four populations harbored two haplotypes (XS, YL, HS, and CK) and 13 populations harbored one haplotype. Hap5 was the most common haplotype in the temperate zone and was shared by 81 individuals in seven populations, while Hap4 was the most common haplotype in the subtropical zone and was shared by 62 individuals of five populations. Hap2, Hap6, Hap7, and Hap8 were private haplotypes in the CK, HS, SNJ, and XS populations, respectively (Table S4). Mt. Daba harbored the most types of haplotypes (six haplotypes), followed by Mt. Qinling (three haplotypes), Mt. Wuling (two haplotypes), and Mt. Taihang (one haplotype) (Figure 4a,b).

The mean value of *N*st (0.964) was significantly higher than that of *G*st (0.904) (*p* < 0.05), indicating that there was a significant phylogeographic structure of *A. griseum*. Eight haplotypes could be roughly clustered into three clades, which showed by the phylogenetic trees constructed consistently by the ML, MP, and NJ methods. Hap1 and Hap2 (central Mt. Daba) were clustered into one clade, Hap3, Hap5, and Hap6 (Mt. Taihang, Mt. Qinling and western Mt. Daba) were clustered into one clade, and Hap4, Hap7, and Hap8 (Mt. Wuling and eastern Mt. Daba) were clustered into one clade (Figure S5).

#### 2.2.5. Haplotype Divergence Time and Demographic History

The haplotype divergence time based on cpDNA indicated that Hap7 was the ancestral of *A. griseum*, which diverged at 25.01 Mya and was only distributed in the SNJ population of Mt. Daba. The divergence time between Hap4 and Hap8 was estimated to be 11.81 Mya. Subsequently, Hap6 diverged at 7.78 Mya, while Hap3 and Hap5 diverged at 6.14 Mya. Finally, the divergence time between Hap1 and Hap2 was estimated to be 2.28 Mya (Figure 4c).

The values of Tajima’s D and Fu and Li’s D* and F* test were calculated as 0.96827, 1.37487, and 1.46863 (*p* > 0.05), respectively. The mismatch distribution of *A. griseum* was multimodal (Figure S6). The above two analyses indicated that the range of *A. griseum* had not undergone rapid contraction or expansion.

### 2.3. Potential Suitable Area in Different Period

A total of 131 coordinate points and eight environmental variables were selected to construct the MaxEnt model, including altitude (alt), mean diurnal range (bio2), temperature seasonality (bio4), min temperature of coldest month (bio6), precipitation of wettest month (bio13), precipitation seasonality (bio15), precipitation of warmest quarter (bio18), and precipitation of coldest quarter (bio19). The average AUC value of the simulation of the suitable area under the current climate was 0.993, indicating that the prediction accuracy and reliability was high.

The response curves of the above eight environmental variables largely conform to normal distribution. The jackknife test of variable importance showed that the following four environmental variables (bio4, bio6, bio13, and bio18) had high gain values, with bio6 being the highest. With an occurrence probability > 0.5 as the threshold, the suitable area of *A. griseum* in bio6 was −8.29~0.27 °C and the peak value was −5.36~−3.72 °C (Figure S7).

**Figure 5:**
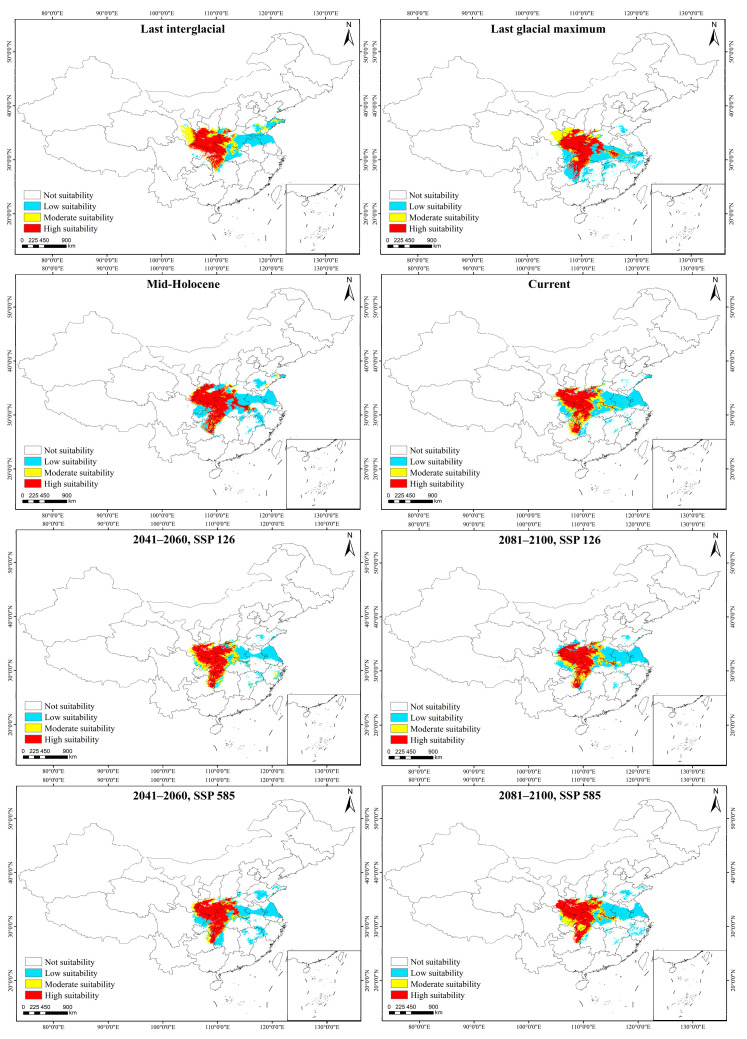
Distribution of suitable habitats for *A. griseum* in different period.

Under the current climate, the suitable habitat of *A. griseum* was 768,888 km^2^, of which the highly suitable habitat was 271,701 km^2^, the moderately suitable habitat was 136,024 km^2^, and the poorly suitable habitat was 361,163 km^2^, accounting for 35.34%, 17.69%, and 46.97%, respectively. Under the historical climate, the suitable habitat of *A. griseum* showed a trend of first increasing and then decreasing. During the Last Interglacial (LIG) period, the suitable habitat of *A. griseum* was the lowest, which was 702,396 km^2^. Subsequently, the suitable habitat increased to 796,528 km^2^ in the Last Glacial Maximum (LGM) period and decreased to 783,698 km^2^ in the Mid-Holocene (MH) period. In future, its suitable habitat will decrease first and then increase, with an overall declining trend. Under the shared socio-economic pathway 126 (SSP126, sustainable development path), the suitable habitat of *A. griseum* will decrease to 647,899 km^2^ from 2041 to 2060 and will increase to 720,538 km^2^ from 2081 to 2100. Similarly, under the SSP585 scenario (conventional development path), the suitable habitat of *A. griseum* will decrease to 694,028 km^2^ from 2041 to 2060 and will increase to 717,308 km^2^ from 2081 to 2100 (Figure 5 and Figure S8).

During the LIG period, the centroid of the suitable habitat of *A. griseum* was Nanyang City, Henan Province (111°53′19.27″ E, 33°21′16.10″ N). During the LGM period, the centroid migrated 224.17 km to the southwest (111°19′47.02″ E, 31°20′35.38″ N). During the MH period, the centroid migrated 116.17 km to the southeast (112°07′52.20″ E, 32°08′21.33″ N). After that, the centroid moved 29.65 km south to Xiangyang City, Hubei Province (112°05′45.02″ E, 31°52′05.03″ N), forming the current pattern of suitable habitat. In the future, the centroid of its suitable habitat will migrate slightly to the northwest in the SSP126 scenario, while the centroid will migrate to northeast in the SSP585 scenario (Figure S9).

## 3. Discussion

### 3.1. Endangered A. griseum Had a Relatively High Level of Genetic Diversity

Genetic diversity of *A. griseum* was relatively high (*H*e = 0.296) compared to the mean value of genetic diversity (*H*e = 0.245) calculated by SNPs of 12 perennial woody plants, such as the widely distributed *A. truncatum* Bunge (*H*e = 0.289) (Table S9) [21,44,45,46,47,48,49,50,51,52,53,54], the genetic diversity of *A. griseum* was relatively high (*H*e = 0.296) (Table S1). Our results were in agreement with the study using SSR markers [43]. Furthermore, compared with other woody plants, such as *Rosa rugosa* Thunb. (*H*t = 0.427) [55], *Cornus sanguinea* L. (*H*t = 0.15) [56], and *Sorbus domestica* L. (*H*t = 0.752) [57], the genetic diversity of *A. griseum* (*H*t = 0.806) based on cpDNA was also relatively high.

The genetic diversity of *A. griseum* based on SNP markers (*H*e = 0.296, *H*o = 0.332, *I* = 0.460, *PIC* = 0.243) (Table S1) was found to be lower than that based on SSR markers (*H*e = 0.727, *H*o = 0.501, *I* = 1.643, *PIC* = 0.678) [43]. This phenomenon has been found in many plants, such as *Cunninghamia lanceolata* (Lamb.) Hook. [47] and *Magnolia sinostellata* P. L. Chiu and Z. H. Chen [58,59]. In general, when analyzing the genetic diversity of the same species, the values of genetic parameters based on SNP markers are lower than those based on SSR markers, even when a larger number of populations and individuals were used [60]. Therefore, it is necessary to compare the data based on the same molecular markers to ensure reliable conclusions when assessing the level of genetic diversity.

### 3.2. Driving Forces for Genetic Diversity and Genetic Differentiation

#### 3.2.1. Orogeny Promoted Haplotype Differentiation by Creating Heterogeneous Microenvironments

The ancestral Hap7 of the SNJ population in Mt. Daba appeared at 25.01 Mya (Figure 4c). The Mid-Himalayan movement occurred at about 30 Mya~20 Mya [61], causing a significant uplift of Mt. Daba. Hap4 and Hap8 diverged at about 11.81 Mya with the uplift of Mt. Daba. At about 15 Mya, Mt. Qinling began to intermittently rapidly uplift [62]. With the uplift of Mt. Qinling, Hap6 emerged in central Mt. Qinling at 7.78 Mya. After that, Hap5, which may be adapted to the semi-humid habitat in eastern Mt. Qinling, and Hap3, which may be adapted to the arid habitat in western Mt. Qinling, were differentiated at 6.14 Mya. Between 5.40 Mya and 2.58 Mya, crustal movement once again led to the sharp uplift of Mt. Qinling and Mt. Daba, which coincided with the divergence of Hap1 and Hap2 (2.78 Mya), suggesting that this geological event drove the differentiation of Hap1 and Hap2. Therefore, it is concluded that the haplotype differentiation process of *A. griseum* bore witness to the uplifting process of the Qinling–Daba Mountains. Furthermore, the variability of altitude induced by orogeny can lead to heterogeneity in the environment, which was a primary factor facilitating the adaptive evolution in plants [63]. The uplift of the Qinling–Daba Mountains caused changes in the microenvironment of *A. griseum*, including elevation, slope, aspect, and even soil thickness and symbiotic biota, which drove it to adapt to the change of ecological niche [32]. *A. griseum* is strongly adaptable to poor site conditions (Figure 1d), which may indicate that it can adapt to the consequences of the uplifting of mountains. Therefore, it is concluded that the heterogeneous habitat caused by orogeny may have been the driving force for haplotype differentiation, which was roughly consistent with species evolution [64]. The Qinling–Daba Mountains are vital sources of high maternal genetic diversity of *A. griseum*, implying that mountains are also cradles of genetic diversity for the species.

#### 3.2.2. Environmental Selection Pressures Led to Adaptive Evolution of *A. griseum*

Based on nDNA, it was estimated that the 17 populations of *A. griseum* were divided into temperate and subtropical groups at 7.42 Mya (Figure 3). Based on cpDNA, the dominant haplotype Hap4 in the subtropical group diverged at 11.81 Mya, while the most common haplotype Hap5 in the temperate group diverged at 6.14 Mya (Figure 4c). The unique evolutionary history of each species plays an important role in shaping its genetic structure. The variability of altitude and distinct climatic gradients within mountainous regions may cause the presence of numerous climatic zones [63]. As the climatic demarcation line between northern and southern China, Mt. Qinling intercepts the cold wave from the north in winter and the tropical moisture from the south in summer, resulting in significant differences in temperature and precipitation between northern and southern China [65]. It leads to a temperate monsoon climate in northern Mt. Qinling and a subtropical monsoon climate in southern Mt. Qinling [66], which further affects the soil types and vegetation distribution in northern and southern Mt. Qinling [67]. Thus, it was speculated that the division of these two groups was caused by the environmental temperature difference of the subtropical and temperate zones to which they belong, caused by the uplifted giant Mt. Qinling [68,69], which can be verified by the results of SDMs.

Based on the KEGG database, the selected genes of Group I of *A. griseum* distributed in the subtropical zone were mainly related to the biosynthesis of flavonoids, such as flavone, flavonol, and isoflavonoid (Table S4). As one type of secondary metabolites involved in plant responses to stress, large amounts of flavonoids can accumulate in plants and help plants to resist a variety of biological and abiotic stresses [70]. In general, different plants respond to diverse biological/abiotic stresses by enriching different flavonoids [70]. The enrichment of flavone and flavonol was used to resist high temperature stress in *Vigna angularis* (Willd.) Ohwi and H. Ohashi [71] and *Solanum lycopersicon* L. [72], while flavone enrichment was used to resist heavy metal stress (Cu, Cd, Pb, etc.) in *Fagopyrum esculentum* Moench [73]. In addition, four genes associated with autophagy were also found. Autophagy plays an important role in plant adaptation to starvation, heat, cold, drought, and other abiotic stresses [74,75,76]. Compared with Group II in the temperate zone, Group I was distributed in a subtropical zone with a higher summer temperature, more abundant precipitation, and higher heavy metal content in the soil [77]. The functions of the above genes implied that *A. griseum* in Group I may have initially evolved a range of genes adapted to heat stress and heavy metal stress in its habitat. However, five genes related to mismatch repair were also identified, indicating that Group I of *A. griseum* may have undergo DNA damage under stresses.

Based on the KEGG database, the selected genes found in *A. griseum* individuals in Group II distributed in the subtropical zone were mainly related to zeatin biosynthesis and endocytosis (Table S4). Zeatin, as a cytokinin, accumulates in large quantities when plants are subjected to salt and drought stress to improve plant resistance [78,79,80]. In addition, endocytosis and inositol phosphate metabolism also play a vital role in tolerating salt stress [81,82], while the phosphatidylinositol signaling system and pyruvate metabolism are also crucial for plant responses to drought stress [83,84]. Compared with Group I in the subtropical zone, Group II was located in a temperate zone with relatively less precipitation and most of the populations were located in saline–alkali land [85]. Therefore, we speculated that *A. griseum* individuals in Group II had evolved a series of genes to help them to adapt to salt and drought stress in their habitat.

The seventeen populations of *A. griseum* were divided into two groups based on nSNPs (Figure 2). However, based on nSSRs, 11 populations were divided into northern (TPZ, LC, and YC), western (NX, TBS, and TS), and southern (YL, WF, SNJ, XS, and CK) groups [43], which was broadly consistent with our results of K = 3. Compared with SSR markers, SNPs have abundant loci and higher genetic stability and can better detect the genetic structure of endangered plants with small populations [20,21]. This situation, in which unlinked SSRs showed better geographical isolation than SNPs, has also been found in other plant species [86]. Compared to the NX population in western Mt. Qinling, selected genes of the TS population in eastern Mt. Qinling were mainly related to photosynthesis, isoflavonoid biosynthesis, the ribosome, and ABC transporters (Table S5). Among them, isoflavonoid biosynthesis and ABC transporters play important roles in plant resistance to drought, salt ion stress, pests, and other physiological processes [87]. In addition, the ribosome biogenesis mechanism favored the maintenance of higher levels of photosynthetic machinery-associated proteins, which maintain gas exchange and photosynthesis under stress [88]. Photosynthesis is also an important process of plant drought tolerance in many plants, such as *Rosa chinensis* Jacq. and *Sorghum bicolor* (L.) Moench, where ribosomes and photosynthetic pathways were involved in the response to drought stress [89]. Hence, we speculated that this subdivision based on nSSRs may be due to the diverse precipitation levels in eastern and western Mt. Qinling.

#### 3.2.3. Mountains Provided Many Glacial Refugia to Maintain Genetic Diversity of *A. griseum*

Neutral test and mismatch analysis showed that the range of *A. griseum* had not undergone rapid contraction or expansion, indicating that its distribution was relatively stable in history (Figure S6). The simulation results based on the MaxEnt model also showed that the suitable habitat of *A. griseum* was relatively stable in history (Figure S8), which was similar to *Dipteronia* Oliv. species [90]. Population bottlenecks can decrease genetic diversity within populations by reducing the effective population size [91,92]. According to our results, *A. griseum* has not experienced significant bottleneck effects in its history, indicating that its stable demographic history has conserved a large amount of genetic variation for *A. griseum*.

Over the course of the Quaternary period, although the range of *A. griseum* was relatively stable at latitude (Figures S6 and S8), its distribution range may have varied in altitude. Since *A. griseum* was only naturally distributed in mid-elevation areas of mountains, we presumed that it was forced to retreat to lower altitudes due to the extremely low temperature during the Quaternary glacial period. However, its samaras mainly disseminate by gravity due to strong lignification [32], so it is difficult to restore to its original high altitudes at interglacial period. Therefore, the four mountains in Central China not only are cradles for the genetic diversity of *A. griseum*, but also provided refugia for it during the Quaternary glacial period.

The CK and XS populations had the highest haplotype diversity (Table S7), while the HS population had a private haplotype (Hap6) and SNJ had a private and ancestral haplotype (Hap8) (Figure 4). In general, populations in glacial refugia have relatively high genetic diversity, ancestral haplotypes, or private haplotypes [93]. Based on maternally inherited cpDNA, the above populations may be the refugia of *A. griseum* during the Quaternary glacial period. Furthermore, since the range of *A. griseum* had not undergone rapid contraction or expansion in history, we concluded that all extant populations of *A. griseum* were its refugia during the Quaternary glacial period. Similarly, the scenario that subtropical China is one of the most important glacial refugia has also been suggested from other studies [94].

#### 3.2.4. High Pollen Flow Among Populations and Out-Breeding Mating System Maintained Genetic Diversity of *A. griseum*

The inbreeding coefficient (*F*is) (Table S1) and artificial pollination experiment [95] indicated that the mating system of *A. griseum* was mainly outcrossing. Inbreeding may lead to heterozygote deficits, decreasing the genetic diversity within populations [96]. Predominantly outcrossed plants possess higher genetic diversity than predominantly inbred plants [97]. In addition, it was also found that the gene flow of *A. griseum* among populations (*N*m = 3.083) was more than 1.0, which helped to counteract the adverse effects of genetic drift [98], thus maintaining the genetic diversity within populations [96]. The mating system dominated by outcrossing and the high level of gene flow among populations are meaningful for the maintenance of high genetic diversity in *A. griseum*.

AMOVA analysis based on nDNA showed that the genetic variation of *A. griseum* was mainly within populations (Table 1), which was similar to the results analyzed by SSR markers. However, the opposite result was obtained based on cpDNA markers (Table 1). This means that the extant spatial genetic structure of *A. griseum* was shaped by a combination of high pollen-mediated gene flow and low seed-mediated gene flow among populations, similar to other plants such as *Quercus aliena* [23] and *Parrotia subaequalis* [24]. *A. griseum*, an insect-pollinated tree, has a large amount of pollen [95], which can lead to a high pollen-mediated gene flow. Furthermore, its seeds are difficult to spread over long distances, which can be elucidated by its biological characteristics. Firstly, as an associated tree species, *A. griseum* is seriously shaded in natural habitats, resulting in fruitlessness.

Secondly, most individuals grow on steep slopes and many seeds are washed into rivers by rain, which makes it difficult for seeds to remain in the soil after falling, resulting in a very small number of seeds kept in the soil seed bank [32]. In general, rivers are important corridors for seed dispersal over long distances for many plants, such as *A. saccharum* Marshall [15] and *A. pycnanthum* K.Koch [99]. However, rivers may also act as obstacles to the seed dispersal of plants, such as *Vitex negundo* L. [100], *Eurycorymbus cavaleriei* (H. Lév.) Rehder and Hand-Mazz. [94], and *Rosa rugosa* [55]. The Yellow River and Yangtze River among the Qinling–Daba Mountains may serve as obstacles to the seed-mediated gene flow of *A. griseum*, because the seeds of *A. griseum* have a high degree of lignification and easily sink to the river bottom after absorbing water. In addition, natural parthenocarpy results in a low full seed rate of only about 50%. Fruits with strong lignification and a harsh habitat lead to difficulties in seed germination and natural regeneration under natural habitats [32], which also limits the seed-mediated flow among populations of *A. griseum*. Herein, we speculated that the striking distribution patterns of genetic variation based on nDNA and cpDNA were the result of high pollen-mediated gene flow and low seed-mediated gene flow.

### 3.3. Conservation Strategy of A. griseum

From the perspective of conservation, populations with high genetic diversity and marginal populations with microecological niche differences to central populations deserve conservation priority [101]. There is a slight natural regeneration in the original populations of *A. griseum*, so it is reasonable to carry out in situ conservation. Priority should be given to the in situ conservation of the CK, HS, SNJ, and XS populations with private/ancestral haplotypes, as well as the marginal populations TS and YL. The above six populations are sufficient to conserve all eight haplotypes detected in the 17 populations of *A. griseum*. The conservation of endangered plants should not only focus on the plant itself, but also on its habitat [102]. Complex interactions of factors, including climate, topography, soil, and related biota, influence the genetic variation of endangered plants [99,103]. Except for the HS population, the other five populations can be efficiently protected because they are located in national nature reserves established between 1982 and 2008. Furthermore, reintroduction is recommended to be conducted through collecting seeds from the preferentially in situ conserved populations to increase the number of individuals in local populations [104].

The simulation results based on the MaxEnt model showed that the highly suitable habitat of *A. griseum* in the Last Interglacial was largely consistent with its current habitat, but expanded southward to northern Guizhou Province in the Last Glacial Maximum, Mid-Holocene, and current climate (Figure S8). However, *A. griseum* is not distributed in Guizhou Province because its seeds are difficult to disseminate over long distances. Jiangsu Province and Anhui Province would be suitable habitats in the future. Ex situ conservation can also be conducted in suitable areas (such as northern Guizhou Province, Anhui Province, and Jiangsu Province) to increase the amount of cultivated individuals for the conservation of its genetic resources.

## 4. Materials and Methods

### 4.1. Sample Sites and DNA Extraction

Leaves from 17 natural populations located in four mountains (Mt. Taihang, Mt. Qinling, Mt. Daba, and Mt. Wuling) of *A. griseum* were collected and rapidly dried in silica gel from 2011 to 2023 (Table 2). The Qinling–Daba Mountains are the main body of the north–south climatic transitional zone of China, spanning subtropical and temperate zones [65]. Furthermore, they are one of the hotspots of biodiversity in China and refugia for many relict, endemic, and endangered species [105,106].

For each population, leaves were sampled from approximately 10~15 individuals with a distance among them around 50 m. Total DNA was isolated from dried leaves using a plant genomic DNA extraction kit (Tiangen Biotech Inc., Beijing, China) and stored at −80 °C. The DNA quality was determined by 1% agarose gel electrophoresis.

### 4.2. SLAF-Seq and SNP Calling of nDNA

The simplified genome sequencing of 170 individuals from 17 natural populations of *A. griseum* was performed by SLAF-seq [107]. The genomic DNA was digested with the Hpy166II + EcoRV restriction enzyme. DNA libraries were constructed and sequenced using the Illumina HiseqTM 2500 sequencing platform (Illumina Inc., San Diego, CA, USA). Clean reads were obtained after the quality assessment and filtration of the sequenced raw reads and mapped to the reference genome of *A. yangbiense* Y. S. Chen and Q. E. Yang [108].

The final reliable SNP marker dataset was developed by SAMtools v1.9 [109] and GATK 3.8 [110]. Plink 1.7 [111] was used to filter out SNP loci that did not meet the integrity of >50% and the minor allele frequency (MAF) of < 0.05 (Dataset 1). Dataset 1 was used for selective sweep analysis. Then, Dataset 2 was obtained by removing SNPs that were significantly deviation from the Hardy–Weinberg equilibrium (HWE, *p* < 0.01) and was used for population genetic analysis [44,112]. In addition, since linkage disequilibrium (LD) may impact the accuracy of PCA and admixture analyses, SNPs with a strong LD (r^2^ < 0.1) were then removed (Dataset 3) and the retained SNPs were used for the PCA and admixture analyses [44,112].

### 4.3. Population Genetic Analysis Based on nDNA

Genetic parameters including *H*e, *H*o, *H*, *I*, *PIC*, *π*, and *F*is were calculated using a perl script developed by Biomarker Technologies Co., Ltd., Beijing, China.

Population structure was analyzed by admixture v1.22 [113]. The number of ancestral populations (K) was set from 1 to 10, determining the best K value based on the minimum value of the cross-validation (CV) error rate. Principal component analysis (PCA) was performed using the smartPCA program in EIGENSOFT v6.0 software [114]. ModelFinder was used to select the best alternative model [115]. With *A. negundo* as an outgroup, the maximum-likelihood (ML) tree was constructed based on MFP model by IQ-TREE 2.2.0 software [116] with 1000 bootstrap replicates. After that, the divergence time was estimated by MCMCtree program in PAML v4.8 software [117] and the calibration divergence time was 29.00 Mya between *A. griseum* and *A. negundo* based on the TimeTree platform (http://www.timetree.org/) (accessed on 14 August 2023).

AMOVA analysis and *F*st were calculated in Arlequin 3.5.2.2 [118]. Gene flow (*N*m) was calculated by the formula *N*m = (1 − *F*st)/4*F*st [119] and the analysis of historical splits or mixtures was performed using TreeMix v1.12 [120]. Mantel correlation analysis between the geographic distance and genetic distance was conducted by the vegan package in R 4.3.1 [121].

The values of *π* and *F*st for each SNP were calculated by VCFtools v0.1.16 [122] using a 100 kb window with a step size of 10 kb for each window. The genomic regions, where the values of *F*st and *π* ratio were all in the top 5%, were defined as the potential selective sweeps. GOseq package of R 4.3.1 [123] was used for GO annotation, while the KOBAS 3.0 program [124] was used for KEGG pathway enrichment analysis.

### 4.4. Complete Chloroplast Genome Sequencing and Development of cpDNA Sequences Primers

Complete chloroplast genome sequences of 26 individuals of 21 locations from four mountains were sequenced by the Illumina Hiseq Platform (Illumina Inc., San Diego, CA, USA). All sequences were aligned to identify mutation sites via applying MAFFT v.7 [125]. Primers were developed at both ends of the genome sequence fragments with more concentrated mutation sites by Primer Premier 6.0 software [126]. A total of 229 individuals from 17 natural populations were amplified by PCR using these primers (Table 1). The PCR amplification system [33] was performed and PCR products were sequenced based on the Sanger sequencing method by ABI PRISM 3730x1 (Thermo Fisher Scientific Inc., Waltham, MA, USA).

### 4.5. Phylogeographic Analysis Based on cpDNA

All sequences were aligned using MAFFT v.7 [125]. The nucleotide diversity (*π*) and haplotype diversity (*H*d) were analyzed by DNAsp v.5.0 [127]. The genetic diversity at the species level (*H*t) and genetic diversity at the population level (*H*s) were calculated by PERMUT [128]. The genetic differentiation coefficients (*G*st and *N*st) were calculated by PERMUT and 1000 random substitution tests were performed. Arlequin 3.5.2.2 software [118] was used to perform AMOVA analysis and calculate the genetic differentiation coefficient (*F*st). The seed-mediated gene flow (*N*m) was calculated by the formula *N*m = (1 − *F*st)/2*F*st.

The haplotype network map was constructed by Network 10.2 software. MEGA-X software [129] was used to reconstruct the ML tree, maximum parsimony tree (MP), and neighbor-joining tree (NJ) of all haplotypes, with *A. truncatum* as an outgroup. The iTOL online platform (https://itol.embl.de/) (accessed on 12 May 2023) was used to view and edit the phylogenetic tree. Haplotype divergence time was estimated by BEAST 1.7.4 software [130]. The Markov chain (MCMC) was adjusted to 5 × 10^7^ generations and sampled every 5000 generations. The calibration divergence time was 28.20 Mya between *A. griseum* and *A. truncatum*, based on the TimeTree platform (http://www.timetree.org/) (accessed on 14 May 2023). The burn-in percentage was set to 1% by using the TreeAnnotation program.

Arlequin 3.5.2.2 software was used to calculate the value of Fu’s Fs and Tajima’s D, and perform the mismatch analysis to predict whether the range of *A. griseum* had experienced significant expansion or contraction in history.

### 4.6. Prediction of Potential Suitable Areas Based on MaxEnt Model

A total of 495 occurrence records of *A. griseum* were obtained based on field investigations, literature, and herbariums, including the Chinese Virtual Herbarium (CVH, http://www.cvh.ac.cn/) (accessed on 18 June 2023) and Global Biodiversity Information Facility (GBIF, http://www.gbif.org/) (accessed on 18 June 2023). ENMTools 1.0 [131] was used to filter the obtained occurrence records to avoid over-fitting of data and affecting the accuracy of prediction results.

A total of 19 climate factors and one altitude factor were downloaded from the WorldClim data website (http://worldclim.org) (accessed on 8 October 2023) at a 2.5 min resolution to simulate the potential suitable areas over different periods: the past (Last Interglacial, Last Glacial Maximum, and Mid-Holocene), present (1970–2000), and future (2041–2061 and 2081–2100). The correlation analysis of 20 environmental variables was carried out using ENMTools 1.0 and the environmental variables with a contribution rate of zero and the ones with a lower contribution rate between two variables with a correlation coefficient of more than 0.80 were removed.

The kuenm package of R 4.3.1 [132] was used to optimize the two parameters of feature class (FC) and regularization multiplier (RM). RM was set to 0.5~4.0, increasing by 0.5 in turn. From 248 parameter combinations, the parameter with the omission rate less than 5% and the lowest delta_AICc value was selected as the optimal model. When the element type was T and the frequency doubling was 1.0, the omission rate was less than 5% and the delta_AICc value was zero.

MaxEnt 3.3 [133] was used to analyze the potential suitable areas of *A. griseum* in different periods with a 10-times repeated operation and the average value being selected as final prediction. Four levels, unsuitable habitat (0.0~0.1), poorly suitable habitat (0.1~0.3), medium suitable habitat (0.3~0.6), and highly suitable habitat (0.6~1.0), were manually set by ArcMap v10.7. The area change and centroid shift of the suitable area in different periods was analyzed by SDMtoolsbox v2.4 tool [134].

## 5. Conclusions

A high level of genetic diversity was found for *A. griseum*. The heterogeneous microenvironment caused by the uplift of the Qinling–Daba Mountains, environmental stress, and high pollen flow among populations were the main driving forces for its high genetic diversity. The four mountains in central China are not only cradles for the genetic diversity of *A. griseum* but also provided glacial refugia for it. Furthermore, the uplifted Qinling–Daba Mountains led to an incongruent genetic structure based on nDNA and cpDNA by high pollen-mediated and low seed-mediated flow. In conclusion, this study represents a new perspective, suggesting that the Qinling–Daba Mountains’ uplifting process has played an important role in plant microevolution through the establishment of maternal genetic structure and proposing a meaningful conservation strategy for *A. griseum*.

## Figures and Tables

**Table 1:** Analysis of molecular variance (AMOVA) analysis of *A. griseum*.

Marker Type	Source of Variation	d.f.	Percentage of Variation	Fixation Index
nDNA	Among populations	16	7.48	*F*st = 0.075
	Within populations	323	92.52	
	Total	339	100.00	
cpDNA	Among populations	16	96.26	*F*st = 0.963
	Within populations	212	3.74	
	Total	228	100.00	

**Table 2:** Sampling information of 17 natural populations of *A. griseum*.

No.	Code	Mountains	Sampling Sites	Latitude (N°)	Longitude (E°)	Altitude (Peak) (m.a.s.l.)	Individuals (cpDNA)
1	YC	Mt. Taihang	Yangcheng county, Shanxi Province	35.27	112.43	534~802 (1994)	15
2	TBS	Mt. Qinling	Taibai mountain, Shaanxi Province	34.08	107.72	1668~1817 (3771)	15
3	HS	Mt. Qinling	Huashan mountain, Shaanxi Province	34.48	110.08	1565~2098 (2155)	15
4	NX	Mt. Qinling	Neixiang county, Henan Province	33.50	111.93	1234~1501 (1380)	13
5	LC	Mt. Qinling	Luanchuan county, Henan Province	33.73	111.63	1066~1388 (2297)	15
6	SX	Mt. Qinling	Song county, Henan Province	34.25	111.87	941~956 (1860)	14
7	TPZ	Mt. Qinling	Taiping town, Xixia county, Henan Province	33.67	111.63	853~902 (2212)	13
8	TS	Mt. Qinling	Tianshui county, Gansu Province	34.23	105.98	1391~1483 (2686)	14
9	CK	Mt. Daba	Chengkou county, Chongqing Municipality	32.10	108.53	1127~1273 (2256)	14
10	GWS	Mt. Daba	Guangwu mountain, Sichuan Province	32.67	106.92	1418~1698 (2507)	10
11	HLS	Mt. Daba	Hualong mountain, Shaanxi Province	32.65	107.67	1517~1732 (2918)	14
12	SNJ	Mt. Daba	Shennongjia nature reserve, Hubei Province	31.46	110.35	1272~1490 (3106)	11
13	XS	Mt. Daba	Xing mountain, Hubei Province	31.31	110.47	1255~1650 (2427)	15
14	ZJJ	Mt. Wuling	Zhangjiajie city, Hunan Province	29.07	110.48	1307~1367 (1519)	13
15	HPS	Mt. Wuling	Huping mountain, Hunan Province	30.05	110.53	1455~1850 (2099)	13
16	YL	Mt. Wuling	Yuanling county, Hunan Province	28.90	110.42	564~826 (1294)	15
17	WF	Mt. Wuling	Wufeng county, Hubei Province	30.15	110.57	1496~1709 (2320)	10
Total							229

## Data Availability

The raw data of SLAF-seq of *Acer griseum* were submitted to NCBI with the accession number PRJNA1071983.

## Supplementary Materials

The supporting information can be downloaded at: https://www.mdpi.com/article/10.3390/ijms26020574/s1.
