# Supplementary material for: Orogeny and High Pollen Flow as Driving Forces for High Genetic Diversity of Endangered Acer griseum (Franch.) Pax Endemic to China
Source: Int J Mol Sci. 2025 Jan 11;26(2):574. doi: 10.3390/ijms26020574 (PMC11765465; doi:10.3390/ijms26020574)
Supplement: Supplementary file 1 [file ijms-26-00574-s001.zip › ijms-3398182-supplementary.pdf]

Supplementary Materials

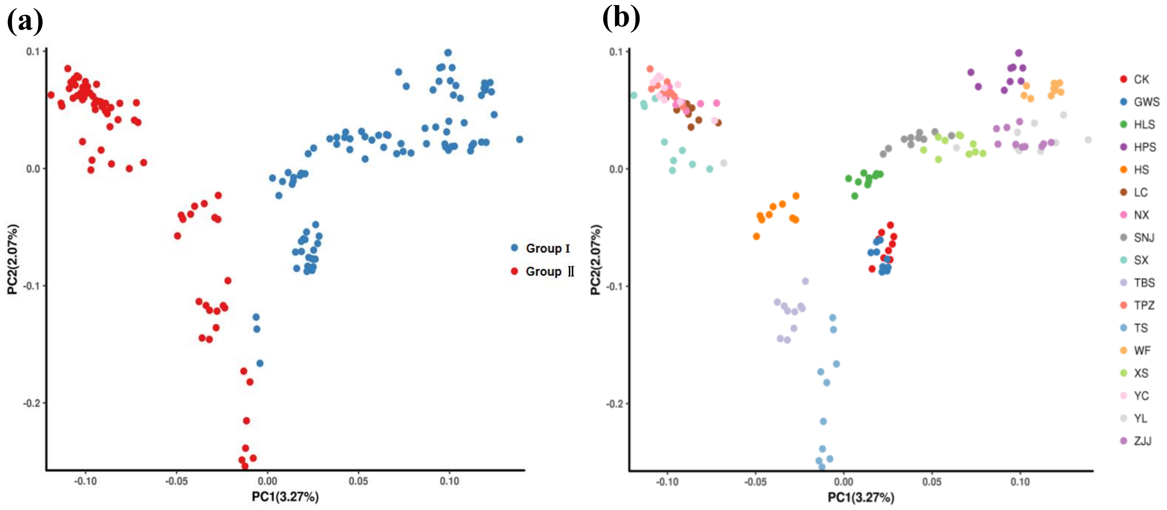

Figure S1. Principal component clustering of *A. griseum*. (a) Principal component clustering of K=2 based on Admixture. (b) Principal component clustering of 17 populations of *A. griseum*.

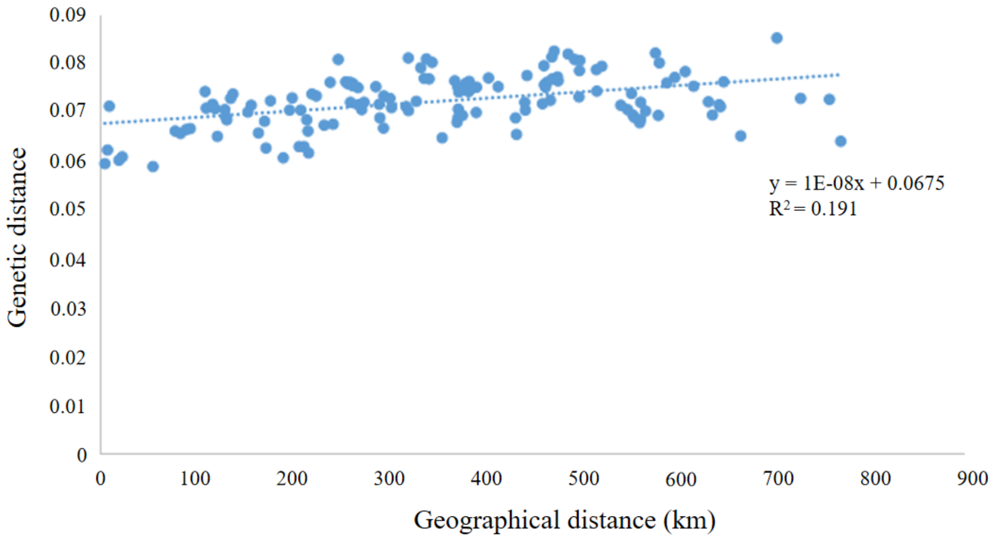

Figure S2. Mantel test on the correlation between genetic distance and geographical distance of *A. griseum*.

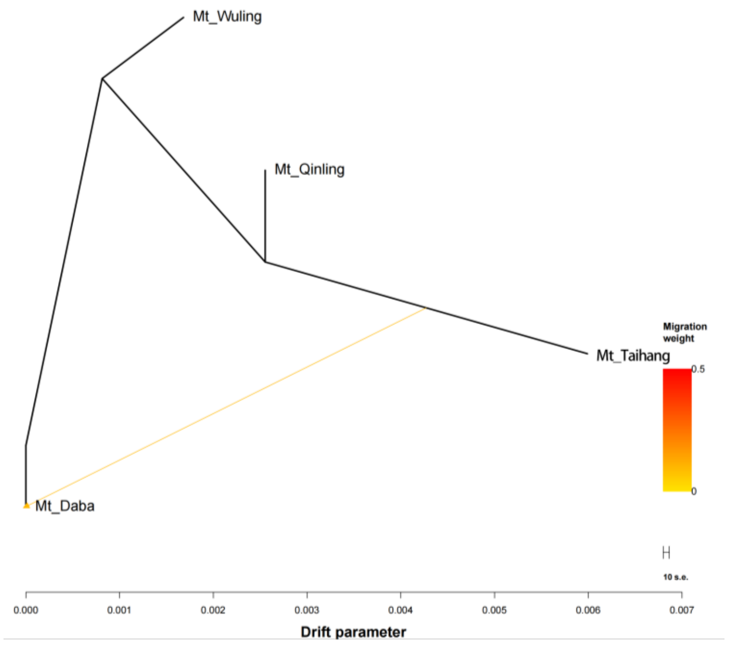

Figure S3. Diagram of Treemix analysis.
Note: The arrow represents the direction of gene flow.

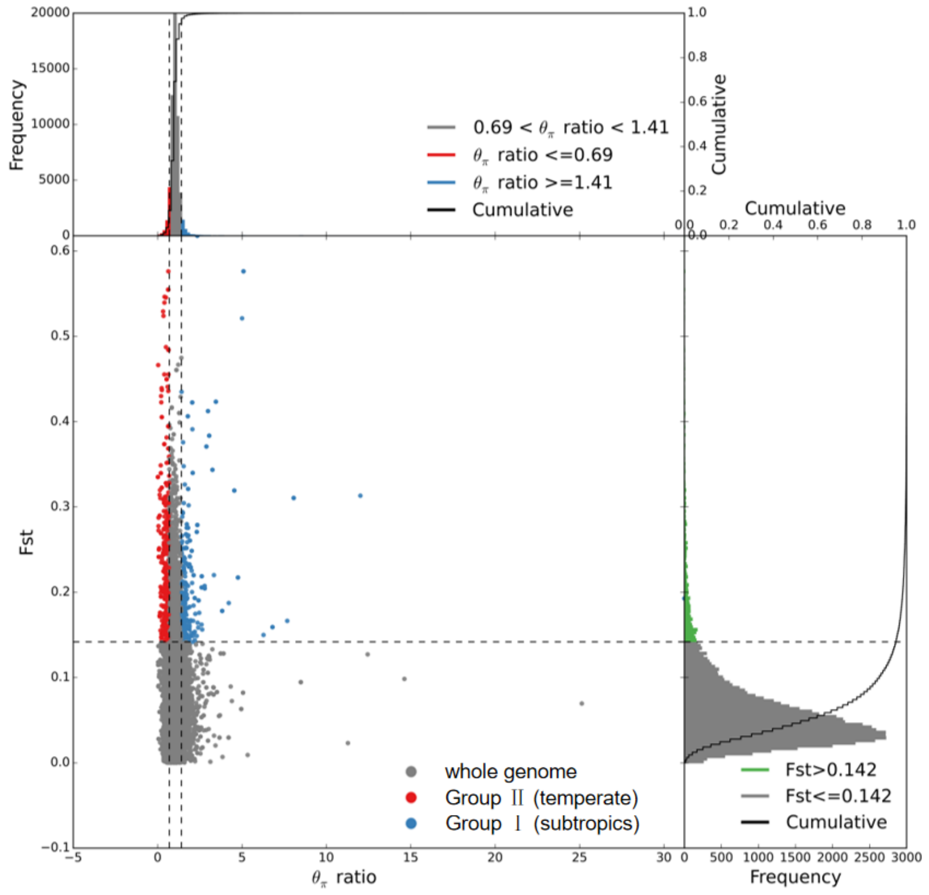

Figure S4. Genomic regions with selective sweeps in Group I and Group II.

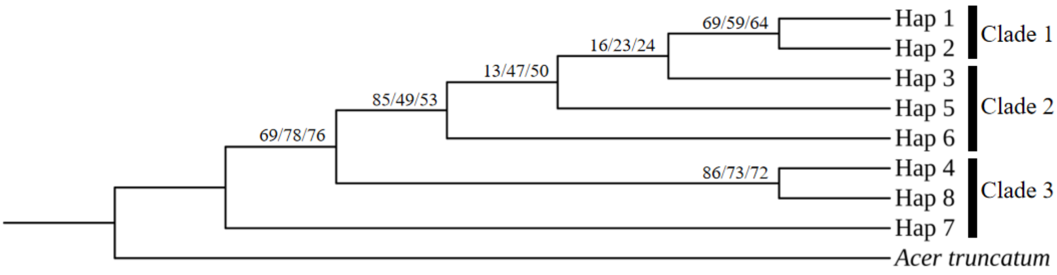

Figure S5. Phylogenetic relationship of eight haplotypes of *A. griseum*.
Note: The values on the branches represent bootstrap value based on ML/MP/NJ.

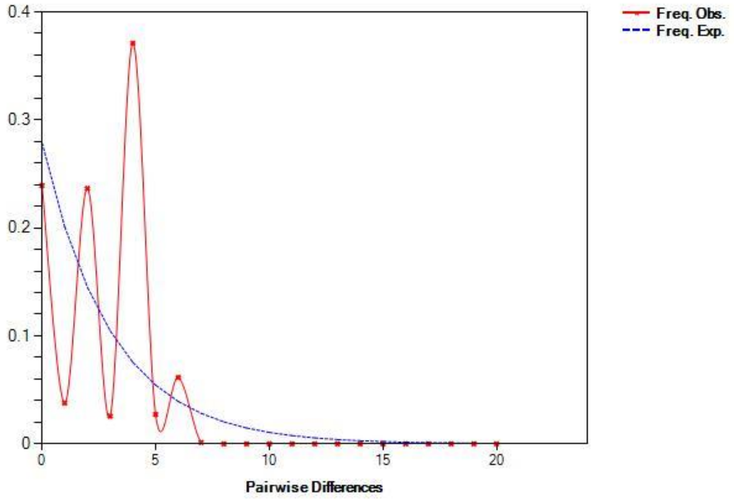

Figure S6. Analysis of mismatch distribution of *A. griseum*.

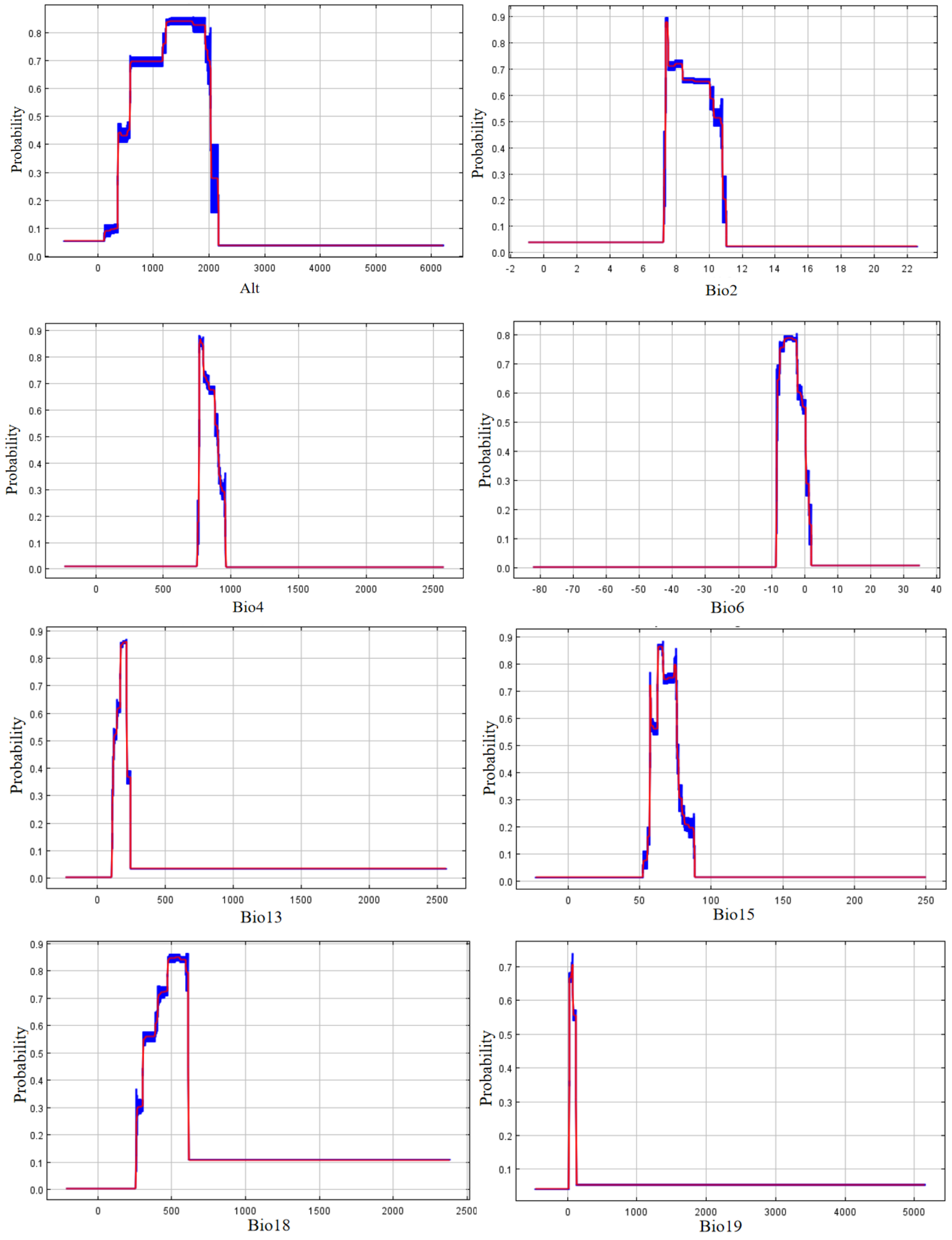

Figure S7. Response curves of eight environmental variables affecting the distribution of suitable areas of *A. griseum*

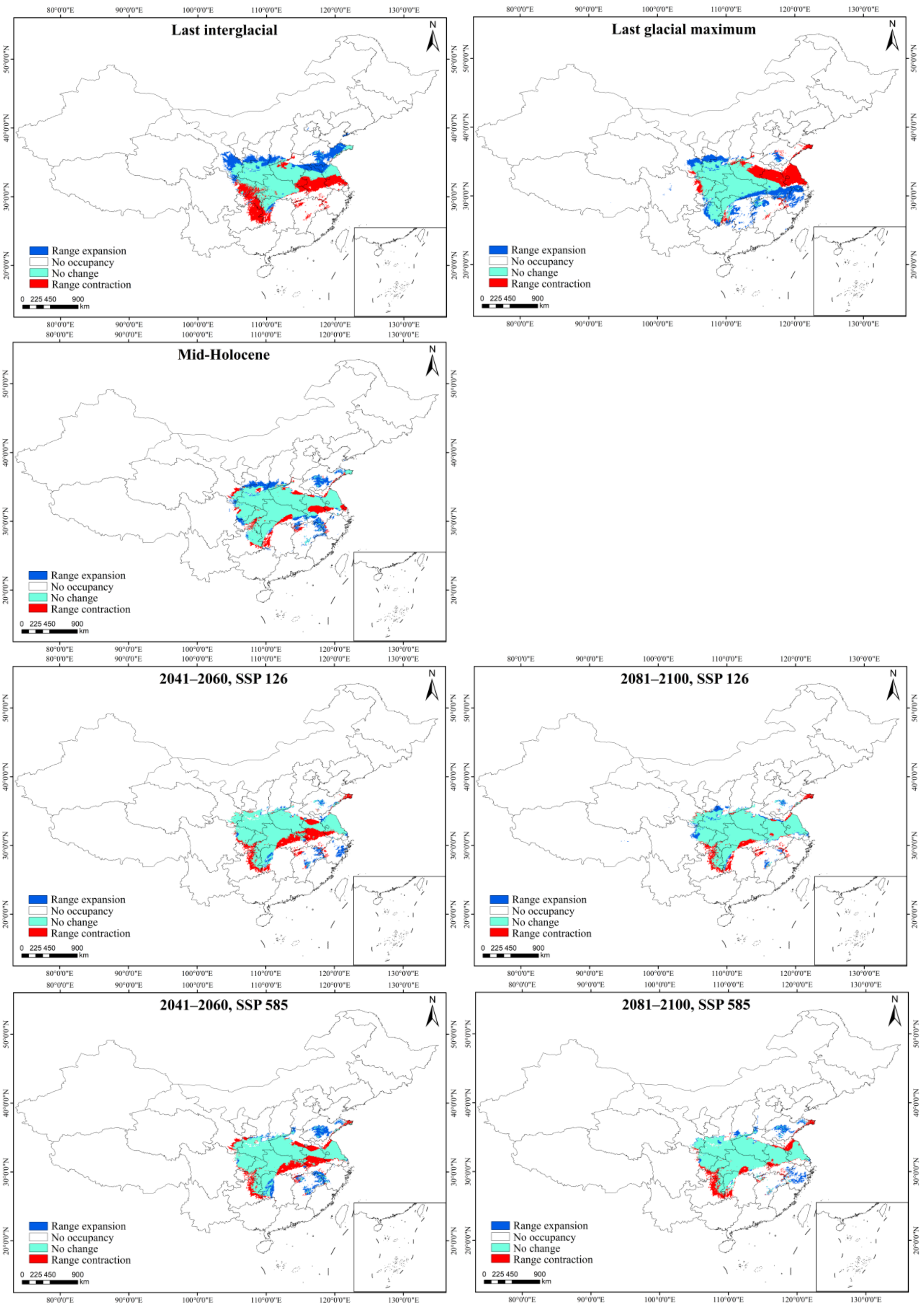

Figure S8. Change of suitable habitats of *A. griseum* at different periods compared with the current climate.

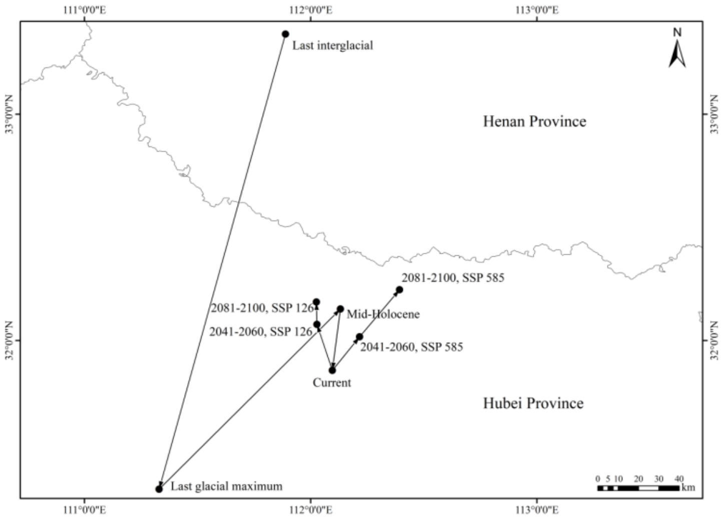

Figure S9. Centroid shift of suitable habitats of *A. griseum* at different periods.

Table S1. Genetic diversity parameters of 17 populations based on nDNA.

Populations	*H*e	*H*o	*H*s	*I*	*PIC*	π	*F*is
YC	0.299	0.336	0.320	0.464	0.245	0.218	-0.124
TBS	0.302	0.353	0.322	0.467	0.247	0.234	-0.169
HS	0.297	0.332	0.318	0.461	0.244	0.235	-0.118
NX	0.293	0.327	0.314	0.457	0.241	0.223	-0.116
LC	0.293	0.327	0.312	0.456	0.241	0.227	-0.116
SX	0.300	0.339	0.321	0.464	0.245	0.219	-0.130
TPZ	0.298	0.336	0.318	0.462	0.244	0.216	-0.128
TS	0.306	0.365	0.325	0.471	0.249	0.229	-0.193
CK	0.296	0.337	0.315	0.460	0.243	0.245	-0.139
GWS	0.291	0.315	0.312	0.454	0.239	0.232	-0.082
HLS	0.289	0.312	0.309	0.451	0.238	0.226	-0.080
SNJ	0.288	0.304	0.310	0.450	0.237	0.217	-0.056
XS	0.286	0.308	0.305	0.448	0.236	0.238	-0.077
ZJJ	0.302	0.337	0.325	0.468	0.247	0.222	-0.116
HPS	0.293	0.320	0.314	0.456	0.241	0.225	-0.092
YL	0.301	0.363	0.323	0.466	0.246	0.252	-0.206
WF	0.296	0.340	0.315	0.460	0.243	0.235	-0.149
Mean value	0.296	0.332	0.316	0.460	0.243	0.229	-0.123

Table S2. Genetic differentiation coefficient (*F*st) and gene flow (*N*m) among four mountains of *A. griseum* based on nDNA

	Mt. Taihang	Mt. Qinling	Mt. Daba	Mt. Wuling
Mt. Taihang		6.274	3.479	2.398
Mt. Qinling	0.038		7.358	3.852
Mt. Daba	0.067	0.033		7.488
Mt. Wuling	0.094	0.061	0.032	

Note: The lower left values indicated *F*st and the upper right values indicated *N*m.

Table S3. GO categories of significant selected genes in Group I and Group II of *A. griseum*.

Group	ID	GO category	Description	p value	geneID
Group I	GO:0048232	Biological process	male gamete generation	0.0004	gene-EZV62_014479/gene-EZV62_014480/gene-EZV62_014481/gene-EZV62_016211
Group I	GO:0055046	Biological process	microgametogenesis	0.0006	gene-EZV62_014479/gene-EZV62_014480/gene-EZV62_014481
Group I	GO:2000024	Biological process	regulation of leaf development	0.0006	gene-EZV62_010403/gene-EZV62_010407/gene-EZV62_025828
Group I	GO:0022412	Biological process	cellular process involved in reproduction in multicellular organism	0.0008	gene-EZV62_014479/gene-EZV62_014480/gene-EZV62_014481/gene-EZV62_016211
Group I	GO:0048609	Biological process	multicellular organismal reproductive process	0.0008	gene-EZV62_014479/gene-EZV62_014480/gene-EZV62_014481/gene-EZV62_016211/gene-EZV62_023302
Group I	GO:0006633	Biological process	fatty acid biosynthetic process	0.0009	gene-EZV62_000731/gene-EZV62_000732/gene-EZV62_000733/gene-EZV62_000734/gene-EZV62_000735 /gene-EZV62_015212/gene-EZV62_017283
Group I	GO:0006914	Biological process	autophagy	0.0010	gene-EZV62_000816/gene-EZV62_004772/gene-EZV62_005549/gene-EZV62_023882
Group I	GO:0007276	Biological process	gamete generation	0.0011	gene-EZV62_014479/gene-EZV62_014480/gene-EZV62_014481/gene-EZV62_016211
Group I	GO:0032504	Biological process	multicellular organism reproduction	0.0011	gene-EZV62_014479/gene-EZV62_014480/gene-EZV62_014481/gene-EZV62_016211/gene-EZV62_023302
Group I	GO:0000003	Biological process	reproduction	0.0024	gene-EZV62_010404/gene-EZV62_010409/gene-EZV62_014479/gene-EZV62_014480/gene-EZV62_014481 /gene-EZV62_016210/gene-EZV62_016211/gene-EZV62_016214/gene-EZV62_018474/gene-EZV62_02330 2/gene-EZV62_024528
Group I	GO:0022414	Biological process	reproductive process	0.0024	gene-EZV62_010404/gene-EZV62_010409/gene-EZV62_014479/gene-EZV62_014480/gene-EZV62_014481 /gene-EZV62_016210/gene-EZV62_016211/gene-EZV62_016214/gene-EZV62_018474/gene-EZV62_02330 2/gene-EZV62_024528

Table S3. GO categories of significant selected genes in Group I and Group II of *A. griseum*.

Group	ID	GO category	Description	p value	geneID
Group I	GO:0051704	Biological process	multi-organism process	0.0032	gene-EZV62_010403/gene-EZV62_010404/gene-EZV62_010409/gene-EZV62_014479/gene-EZV62_014480/gene-EZV62_014481/gene-EZV62_014887/gene-EZV62_016211/gene-EZV62_023882/gene-EZV62_025243/gene-EZV62_025828
Group I	GO:0045017	Biological process	glycerolipid biosynthetic process	0.0035	gene-EZV62_020391/gene-EZV62_020392/gene-EZV62_020394
Group I	GO:0009889	Biological process	regulation of biosynthetic process	0.0054	gene-EZV62_004784/gene-EZV62_007818/gene-EZV62_010050/gene-EZV62_010964/gene-EZV62_013604/gene-EZV62_014479/gene-EZV62_014480/gene-EZV62_014481/gene-EZV62_015771/gene-EZV62_017288/gene-EZV62_018316/gene-EZV62_023302/gene-EZV62_025828
Group I	GO:0044703	Biological process	multi-organism reproductive process	0.0059	gene-EZV62_014479/gene-EZV62_014480/gene-EZV62_014481/gene-EZV62_016211
Group I	GO:0043043	Biological process	peptide biosynthetic process	0.0062	gene-EZV62_010414/gene-EZV62_020524/gene-EZV62_027839
Group I	GO:0043604	Biological process	amide biosynthetic process	0.0071	gene-EZV62_010414/gene-EZV62_020524/gene-EZV62_027839
Group I	GO:0019953	Biological process	sexual reproduction	0.0075	gene-EZV62_014479/gene-EZV62_014480/gene-EZV62_014481/gene-EZV62_016211
Group I	GO:0032502	Biological process	developmental process	0.0093	gene-EZV62_010050/gene-EZV62_010403/gene-EZV62_010404/gene-EZV62_013341/gene-EZV62_014479/gene-EZV62_014480/gene-EZV62_014481/gene-EZV62_016210/gene-EZV62_016211/gene-EZV62_016214/gene-EZV62_018474/gene-EZV62_022743/gene-EZV62_023305/gene-EZV62_023882/gene-EZV62_024528
Group I	GO:0007275	Biological process	multicellular organism development	0.0097	gene-EZV62_010050/gene-EZV62_010403/gene-EZV62_010404/gene-EZV62_013341/gene-EZV62_014479/gene-EZV62_014480/gene-EZV62_014481/gene-EZV62_015819/gene-EZV62_016210/gene-EZV62_016214/gene-EZV62_018474/gene-EZV62_024528/gene-EZV62_024529

Table S3. GO categories of significant selected genes in Group I and Group II of *A. griseum*.

Group	ID	GO category	Description	p value	geneID
Group I	GO:0016747	Molecular function	transferase activity, transferring acyl groups other than amino-acyl groups	1.37E-15	gene-EZV62_000731/gene-EZV62_000732/gene-EZV62_000733/gene-EZV62_000734/gene-EZV62_000735/gene-EZV62_013606/gene-EZV62_013968/gene-EZV62_013969/gene-EZV62_013971/gene-EZV62_013972/gene-EZV62_013973/gene-EZV62_013974/gene-EZV62_013976/gene-EZV62_013977/gene-EZV62_013978/gene-EZV62_013979/gene-EZV62_013980/gene-EZV62_013981/gene-EZV62_020523/gene-EZV62_020525/gene-EZV62_020526/gene-EZV62_020527/gene-EZV62_020528/gene-EZV62_020529/gene-EZV62_020530
Group I	GO:0015299	Molecular function	solute:proton antiporter activity	0.0005	gene-EZV62_014966/gene-EZV62_018327/gene-EZV62_025908/gene-EZV62_025916/gene-EZV62_025917
Group I	GO:0140110	Molecular function	transcription regulator activity	0.0015	gene-EZV62_010050/gene-EZV62_013604/gene-EZV62_014479/gene-EZV62_014480/gene-EZV62_014481/gene-EZV62_015771/gene-EZV62_017288/gene-EZV62_018316/gene-EZV62_023302/gene-EZV62_025828
Group I	GO:0004144	Molecular function	diacylglycerol O-acyltransferase activity	0.0041	gene-EZV62_020391/gene-EZV62_020392/gene-EZV62_020394
Group II	GO:0042542	Biological process	response to hydrogen peroxide	0.0002	gene-EZV62_001409/gene-EZV62_002800/gene-EZV62_002802/gene-EZV62_015147
Group II	GO:0009628	Biological process	response to abiotic stimulus	0.0003	gene-EZV62_001409/gene-EZV62_002800/gene-EZV62_002802/gene-EZV62_004819/gene-EZV62_008642/gene-EZV62_012279/gene-EZV62_015147/gene-EZV62_015805/gene-EZV62_018371/gene-EZV62_022246/gene-EZV62_023839/gene-EZV62_024564/gene-EZV62_028226
Group II	GO:0000302	Biological process	response to reactive oxygen species	0.0003	gene-EZV62_001409/gene-EZV62_002800/gene-EZV62_002802/gene-EZV62_004819/gene-EZV62_015147
Group II	GO:0002218	Biological process	activation of innate immune response	0.0003	gene-EZV62_017307/gene-EZV62_019597/gene-EZV62_024564
Group II	GO:0002253	Biological process	activation of immune response	0.0004	gene-EZV62_017307/gene-EZV62_019597/gene-EZV62_024564

Table S3. GO categories of significant selected genes in Group I and Group II of *A. griseum*.

Group	ID	GO category	Description	p value	geneID
Group II	GO:0045089	Biological process	positive regulation of innate immune response	0.0009	gene-EZV62_017307/gene-EZV62_019597/gene-EZV62_024564
Group II	GO:0048511	Biological process	rhythmic process	0.0009	gene-EZV62_012279/gene-EZV62_015147/gene-EZV62_018371
Group II	GO:0031324	Biological process	negative regulation of cellular metabolic process	0.0010	gene-EZV62_004751/gene-EZV62_008642/gene-EZV62_012298/gene-EZV62_018498/gene-EZV62_023049/gene-EZV62_023050/gene-EZV62_023839
Group II	GO:0042221	Biological process	response to chemical	0.0017	gene-EZV62_001409/gene-EZV62_002800/gene-EZV62_002802/gene-EZV62_004819/gene-EZV62_010267/gene-EZV62_012279/gene-EZV62_012298/gene-EZV62_015147/gene-EZV62_015435/gene-EZV62_015808/gene-EZV62_018498/gene-EZV62_022246/gene-EZV62_023839/gene-EZV62_024564/gene-EZV62_028226
Group II	GO:0002684	Biological process	positive regulation of immune system process	0.0020	gene-EZV62_017307/gene-EZV62_019597/gene-EZV62_024564
Group II	GO:0007623	Biological process	circadian rhythm	0.0020	gene-EZV62_012279/gene-EZV62_015147/gene-EZV62_018371
Group II	GO:0031349	Biological process	positive regulation of defense response	0.0020	gene-EZV62_017307/gene-EZV62_019597/gene-EZV62_024564
Group II	GO:0050778	Biological process	positive regulation of immune response	0.0020	gene-EZV62_017307/gene-EZV62_019597/gene-EZV62_024564
Group II	GO:0044282	Biological process	small molecule catabolic process	0.0023	gene-EZV62_004827/gene-EZV62_004829/gene-EZV62_015435
Group II	GO:0090351	Biological process	seedling development	0.0036	gene-EZV62_012279/gene-EZV62_015434/gene-EZV62_015435
Group II	GO:0009408	Biological process	response to heat	0.0038	gene-EZV62_002800/gene-EZV62_002802/gene-EZV62_004819/gene-EZV62_022246/gene-EZV62_028226
Group II	GO:0044248	Biological process	cellular catabolic process	0.0042	gene-EZV62_004827/gene-EZV62_004829/gene-EZV62_008642/gene-EZV62_012298/gene-EZV62_015435

Table S3. GO categories of significant selected genes in Group I and Group II of *A. griseum*.

Group	ID	GO category	Description	p value	geneID
					/gene-EZV62_015470/gene-EZV62_015808/gene-EZV62_022246/gene-EZV62_028226
Group II	GO:0009266	Biological process	response to temperature stimulus	0.0044	gene-EZV62_002800/gene-EZV62_002802/gene-EZV62_004819/gene-EZV62_022246/gene-EZV62_023839 /gene-EZV62_028226
Group II	GO:0009892	Biological process	negative regulation of metabolic process	0.0044	gene-EZV62_004751/gene-EZV62_008642/gene-EZV62_012298/gene-EZV62_018498/gene-EZV62_023049 /gene-EZV62_023050/gene-EZV62_023839
Group II	GO:0080134	Biological process	regulation of response to stress	0.0045	gene-EZV62_015147/gene-EZV62_017307/gene-EZV62_018498/gene-EZV62_019597/gene-EZV62_024564
Group II	GO:0031347	Biological process	regulation of defense response	0.0047	gene-EZV62_017307/gene-EZV62_018498/gene-EZV62_019597/gene-EZV62_024564
Group II	GO:0042594	Biological process	response to starvation	0.0052	gene-EZV62_001409/gene-EZV62_015147/gene-EZV62_023839
Group II	GO:0045088	Biological process	regulation of innate immune response	0.0052	gene-EZV62_017307/gene-EZV62_019597/gene-EZV62_024564
Group II	GO:0009056	Biological process	catabolic process	0.0052	gene-EZV62_004827/gene-EZV62_004829/gene-EZV62_008642/gene-EZV62_012298/gene-EZV62_015435 /gene-EZV62_015470/gene-EZV62_015808/gene-EZV62_022246/gene-EZV62_028226
Group II	GO:0009636	Biological process	response to toxic substance	0.0053	gene-EZV62_001409/gene-EZV62_002800/gene-EZV62_002802/gene-EZV62_015147/gene-EZV62_028226
Group II	GO:1901575	Biological process	organic substance catabolic process	0.0053	gene-EZV62_004827/gene-EZV62_004829/gene-EZV62_008642/gene-EZV62_012298/gene-EZV62_015435 /gene-EZV62_015470/gene-EZV62_015808/gene-EZV62_022246
Group II	GO:0031323	Biological process	regulation of cellular metabolic process	0.0054	gene-EZV62_004751/gene-EZV62_008642/gene-EZV62_010267/gene-EZV62_012082/gene-EZV62_012298 /gene-EZV62_015805/gene-EZV62_018371/gene-EZV62_018421/gene-EZV62_018498/gene-EZV62_02212 0/gene-EZV62_023049/gene-EZV62_023050/gene-EZV62_023839
Group II	GO:0010033	Biological process	response to organic substance	0.0055	gene-EZV62_001409/gene-EZV62_002800/gene-EZV62_002802/gene-EZV62_010267/gene-EZV62_012279 /gene-EZV62_012298/gene-EZV62_015808/gene-EZV62_018498/gene-EZV62_022246/gene-EZV62_02383 9/gene-EZV62_028226

Table S3. GO categories of significant selected genes in Group I and Group II of *A. griseum*.

Group	ID	GO category	Description	p value	geneID
Group II	GO:0031327	Biological process	negative regulation of cellular biosynthetic process	0.0056	gene-EZV62_012298/gene-EZV62_018498/gene-EZV62_023049/gene-EZV62_023050/gene-EZV62_023839
Group II	GO:0009890	Biological process	negative regulation of biosynthetic process	0.0065	gene-EZV62_012298/gene-EZV62_018498/gene-EZV62_023049/gene-EZV62_023050/gene-EZV62_023839
Group II	GO:0031326	Biological process	regulation of cellular biosynthetic process	0.0070	gene-EZV62_010267/gene-EZV62_012082/gene-EZV62_012298/gene-EZV62_015805/gene-EZV62_018371/gene-EZV62_018421/gene-EZV62_018498/gene-EZV62_022120/gene-EZV62_023049/gene-EZV62_023050/gene-EZV62_023839
Group II	GO:0031667	Biological process	response to nutrient levels	0.0071	gene-EZV62_001409/gene-EZV62_015147/gene-EZV62_023839
Group II	GO:0062014	Biological process	negative regulation of small molecule metabolic process	0.0071	gene-EZV62_012298/gene-EZV62_023049/gene-EZV62_023050
Group II	GO:0042493	Biological process	response to drug	0.0075	gene-EZV62_001409/gene-EZV62_002800/gene-EZV62_002802/gene-EZV62_015147/gene-EZV62_015435
Group II	GO:0009889	Biological process	regulation of biosynthetic process	0.0080	gene-EZV62_010267/gene-EZV62_012082/gene-EZV62_012298/gene-EZV62_015805/gene-EZV62_018371/gene-EZV62_018421/gene-EZV62_018498/gene-EZV62_022120/gene-EZV62_023049/gene-EZV62_023050/gene-EZV62_023839
Group II	GO:0090698	Biological process	post-embryonic plant morphogenesis	0.0087	gene-EZV62_012298/gene-EZV62_015244/gene-EZV62_022246
Group II	GO:0048523	Biological process	negative regulation of cellular process	0.0091	gene-EZV62_004751/gene-EZV62_008642/gene-EZV62_012298/gene-EZV62_018421/gene-EZV62_018498/gene-EZV62_023049/gene-EZV62_023050/gene-EZV62_023839
Group II	GO:0006099	Biological process	tricarboxylic acid cycle	0.0095	gene-EZV62_008069/gene-EZV62_023835/gene-EZV62_023836
Group II	GO:0006950	Biological process	response to stress	0.0099	gene-EZV62_001409/gene-EZV62_002800/gene-EZV62_002802/gene-EZV62_004819/gene-EZV62_012082/gene-EZV62_015147/gene-EZV62_015808/gene-EZV62_017307/gene-EZV62_018498/gene-EZV62_01959

Table S3. GO categories of significant selected genes in Group I and Group II of *A. griseum*.

Group	ID	GO category	Description	p value	geneID
					7/gene-EZV62_022246/gene-EZV62_023839/gene-EZV62_024564/gene-EZV62_028226
Group II	GO:0030880	Cellular component	RNA polymerase complex	0.0019	gene-EZV62_004754/gene-EZV62_012082/gene-EZV62_015805
Group II	GO:0008194	Molecular function	UDP-glycosyltransferase activity	0.0003	gene-EZV62_002786/gene-EZV62_002787/gene-EZV62_002790/gene-EZV62_002791/gene-EZV62_002792/gene-EZV62_002793/gene-EZV62_002794/gene-EZV62_002795/gene-EZV62_002796/gene-EZV62_002799/gene-EZV62_002801/gene-EZV62_023166/gene-EZV62_023167
Group II	GO:0016829	Molecular function	lyase activity	0.0038	gene-EZV62_004827/gene-EZV62_004829/gene-EZV62_007799
Group II	GO:0008080	Molecular function	N-acetyltransferase activity	0.0041	gene-EZV62_012296/gene-EZV62_015805/gene-EZV62_020431/gene-EZV62_020436

Table S4. Significant selected genes in Group I and Group II of *A. griseum* based on KEGG database.

Group	ID	Description	p value	geneID
Group I	ko00944	Flavone and flavonol biosynthesis	0.0000	gene-EZV62_020523/gene-EZV62_020525/gene-EZV62_020526/gene-EZV62_020527/gene-EZV62_020528/gene-EZV62_020529/gene-EZV62_020530
Group I	ko00943	Isoflavonoid biosynthesis	0.0002	gene-EZV62_020523/gene-EZV62_020525/gene-EZV62_020526/gene-EZV62_020527/gene-EZV62_020528/gene-EZV62_020529/gene-EZV62_020530
Group I	ko00062	Fatty acid elongation	0.0009	gene-EZV62_000731/gene-EZV62_000732/gene-EZV62_000733/gene-EZV62_000734/gene-EZV62_000735
Group I	ko03430	Mismatch repair	0.0033	gene-EZV62_017289/gene-EZV62_017290/gene-EZV62_017291/gene-EZV62_017292/gene-EZV62_023892
Group I	ko04136	Autophagy - other	0.0074	gene-EZV62_004772/gene-EZV62_005549/gene-EZV62_010965/gene-EZV62_023882
Group II	ko00908	Zeatin biosynthesis	0.0000	gene-EZV62_002786/gene-EZV62_002787/gene-EZV62_002788/gene-EZV62_002789/gene-EZV62_002791/gene-EZV62_002792/gene-EZV62_002793/gene-EZV62_002794/gene-EZV62_002795/gene-EZV62_002796/gene-EZV62_002799/gene-EZV62_002801
Group II	ko04070	Phosphatidylinositol signaling system	0.0010	gene-EZV62_015396/gene-EZV62_020429/gene-EZV62_020432/gene-EZV62_020434/gene-EZV62_020435/gene-EZV62_020437/gene-EZV62_020439
Group II	ko04144	Endocytosis	0.0010	gene-EZV62_004750/gene-EZV62_011299/gene-EZV62_012407/gene-EZV62_012732/gene-EZV62_015425/gene-EZV62_016406/gene-EZV62_020429/gene-EZV62_020432/gene-EZV62_020434/gene-EZV62_020435/gene-EZV62_020437/gene-EZV62_020439/gene-EZ

Table S4. Significant selected genes in Group I and Group II of *A. griseum* based on KEGG database.

Group	ID	Description	p value	geneID
				V62_020444
Group II	ko00562	Inositol phosphate metabolism	0.0021	gene-EZV62_007798/gene-EZV62_020429/gene-EZV62_020432/gene-EZV62_020434/gene-EZV62_020435/gene-EZV62_020437/gene-EZV62_020439
Group II	ko00620	Pyruvate metabolism	0.0038	gene-EZV62_004827/gene-EZV62_004829/gene-EZV62_008069/gene-EZV62_017299/gene-EZV62_019596/gene-EZV62_023835/gene-EZV62_023836

Table S5. Significant selected genes in TS population and NX population of *A. griseum* based on KEGG database.

Population	ID	Description	p value	geneID
TS	ko00195	Photosynthesis	2.30E-07	gene-EZV62_000638/gene-EZV62_014710/gene-EZV62_014711/gene-EZV62_020534/gene-EZV62_027815/gene-EZV62_027818/gene-EZV62_027819/gene-EZV62_027820/gene-EZV62_027821/gene-EZV62_027822/gene-EZV62_027824/gene-EZV62_027825/gene-EZV62_027827/gene-EZV62_027828
TS	ko00943	Isoflavonoid biosynthesis	0.0012	gene-EZV62_013927/gene-EZV62_013928/gene-EZV62_018213/gene-EZV62_027937/gene-EZV62_027938/gene-EZV62_027939/gene-EZV62_027940/gene-EZV62_027941
TS	ko03010	Ribosome	0.0058	gene-EZV62_000289/gene-EZV62_004701/gene-EZV62_014292/gene-EZV62_014348/gene-EZV62_015245/gene-EZV62_016938/gene-EZV62_017789/gene-EZV62_017855/gene-EZV62_020128/gene-EZV62_020131/gene-EZV62_020136/gene-EZV62_020294/gene-EZV62_021166/gene-EZV62_021689/gene-EZV62_022863/gene-EZV62_027829/gene-EZV62_027830/gene-EZV62_027831/gene-EZV62_027833/gene-EZV62_027834
TS	ko02010	ABC transporters	0.0092	gene-EZV62_001850/gene-EZV62_001851/gene-EZV62_010196/gene-EZV62_010198/gene-EZV62_010199/gene-EZV62_016904/gene-EZV62_016905/gene-EZV62_016906/gene-EZV62_019436/gene-EZV62_020309/gene-EZV62_022966/gene-EZV62_023854/gene-EZV62_030055
NX	ko00062	Fatty acid elongation	0.0014	gene-EZV62_011541/gene-EZV62_011543/gene-EZV62_011544/gene-EZV62_011547

Table S6. Three pairs of cpDNA primers

Forward primer	Reverse primer	Tm
AGTTCAAATCTGGGTGTCGC	CCAATCAATCTGAAATAATCGC	58℃
AATAGGGATACCTCCCAACA	TCCACAATAATGAAACAAATCAGGA	58℃
AGATTTGTGCCAGGGGTTCA	TCCTCGCTGGTCATACAACT	58℃

Table S7. Haplotype diversity of 17 populations of *A. griseum* based on cpDNA.

Mountain	Population code	Number of individuals	Haplotype (number of individuals)	*H*d	π
Mt. Taiang	YC	15	Hap 5(15)	0.000	0.00000
Total		15	Hap 5(15)	0.000	0.00000
Mt. Qinling	TBS	15	Hap 3(15)	0.000	0.00000
	HS	15	Hap 5(10), Hap 6(5)	0.476	0.00022
	NX	13	Hap 5(13)	0.000	0.00000
	LC	15	Hap 5(15)	0.000	0.00000
	SX	14	Hap 5(14)	0.000	0.00000
	TPZ	13	Hap 5(13)	0.000	0.00000
	TS	14	Hap 3(14)	0.000	0.00000
Total		99	Hap 3(29), Hap 5(65), Hap 6(5)	0.485	0.00018
Mt. Daba	CK	14	Hap 1(11), Hap 2(3)	0.363	0.00016
	GWS	10	Hap 3(10)	0.000	0.00000
	HLS	14	Hap 1(14)	0.000	0.00000
	SNJ	11	Hap 7(11)	0.000	0.00000
	XS	15	Hap 4(12), Hap 8(3)	0.343	0.00016
Total		64	Hap 1(25), Hap 2(3), Hap 3(10), Hap 4(12), Hap 7(11), Hap 8(3)	0.766	0.00144
Mt. Wuling	ZJJ	13	Hap 4(13)	0.000	0.00000
	HPS	13	Hap 4(13)	0.000	0.00000
	YL	15	Hap 4(14), Hap 5(1)	0.133	0.00024
	WF	10	Hap 4(10)	0.000	0.00000
Total		51	Hap 4(50), Hap 5(1)	0.039	0.00007

Table S8. Genetic differentiation coefficient (*F*st) and seed-mediate gene flow (*N*m) among four mountains of *A. griseum* based on cpDNA

	Mt. Taihang	Mt. Qinling	Mt. Daba	Mt. Wuling
Mt. Taihang		1.097	0.472	0.010
Mt. Qinling	0.313		0.925	0.077
Mt. Daba	0.514	0.351		0.553
Mt. Wuling	0.980	0.867	0.475	

Note: The lower left values indicated *F*st and the upper right values indicated *N*m.

Table S9. Genetic diversity of 12 perennial woody plants based on genomic SNPs

Species	Sequencing method	Mean value of *He*	Number of populations / locations (individuals)	Reference
Acer truncatum Bunge	Re-sequencing	0.289	13 (130)	[44]
Carpinus oblongifolia (Hu) Hu & W. C. Cheng	SLAF	0.289	1 (40)	[45]
Cinnamomum camphora (L.) J. Presl	GBS	0.352	40 (171)	[46]
Cunninghamia lanceolata (Lamb.) Hook.	SLAF	0.278	1 (50)	[47]
Elaeis guineensis Jacq.	SLAF	0.318	21 (200)	[48]
Euscaphis japonica (Thunb. ex Roem. & Schult.) Kanitz	SLAF	0.335	12 (94)	[49]
Ficus hirta Vahl	dd-RAD	0.280	17 (141)	[50]
Jubaea chilensis (Molina) Baill.	GBS	0.024	6 (140)	[51]
Litsea populifolia (Hemsl.) Gamble	GBS	0.246	7 (84)	[52]
Paeonia ludlowii (Stern & G. Taylor) J. J. Li & D. Z. Chen	RAD	0.032	4 (40)	[21]
Quercus shumardii Buckley	SLAF	0.190	6 (30)	[53]
Tetraena mongolica Maxim.	GBS	0.348	8 (120)	[54]
Mean value		0.248		
